# Associations between lifestyle habits, environmental factors and respiratory diseases: a cross-sectional study from southwest China

**DOI:** 10.3389/fpubh.2025.1513926

**Published:** 2025-03-05

**Authors:** Hengyu Su, Huifang Xie

**Affiliations:** School of Public Health, Xinjiang Medical University, Ürümqi, China

**Keywords:** lifestyle habits, environmental factors, respiratory diseases, cross-sectional study, subgroup interaction

## Abstract

**Background:**

Numerous studies have demonstrated that lifestyle habits and environmental factors influence the incidence and progression of respiratory diseases. However, there is a paucity of similar research conducted in southwest China.

**Objective:**

This study aims to investigate the prevalence and primary influencing factors of respiratory diseases among residents in a specific region of southwest China, and to identify vulnerable populations.

**Method:**

From February 2024 to May 2024, a multi-stage stratified random sampling method was employed in a specific region of southwest China. Three monitoring points were randomly selected from six jurisdictions within this region, resulting in the collection of relevant information from a total of 4,507 residents through offline interviews. Lasso-logistic regression was conducted using R version 4.3.0 to develop a nomogram for estimating disease probabilities. Interaction analysis was performed with gender and age group serving as grouping variables, while other dimensional factors were utilized as analysis variables.

**Result:**

A total of 4,507 respondents participated in this study, of whom 956 (21.21%) were identified as sick. The older adult group (>65 years) exhibited the highest prevalence (30.3%). Results from the Lasso-logistic model indicated that current smoking, alcohol abuse, passive smoking, coupled with poor indoor and outdoor environments were significant risk factors. Additionally, a history of respiratory disease, a family history of respiratory issues, negative emotions, and high stress levels may also contribute to the risk of the disease. Protective factors identified include regular exercise, adequate indoor lighting, frequent ventilation, and regular disinfection practices. The nomogram developed in this study demonstrated good discrimination, calibration, and clinical efficacy. Multiplicative interaction analysis indicated that gender and age group exhibited varying degrees of interaction with factors such as smoking, passive smoking, alcohol abuse, regular exercise, household smoke, house disinfection, dust mites, history of respiratory allergies, use of velvet products, and family history of respiratory conditions. Notably, females, adolescents, and the older adult were identified as particularly susceptible and at-risk groups for these interactions.

**Conclusion:**

The prevalence of respiratory diseases is notably higher among the permanent population in southwest China. High-risk lifestyles, coupled with poor indoor and outdoor environments, pose particularly significant threats to women, adolescents, and the older adult. Consequently, improving living habits, renovating aging communities, enhancing the quality of the living environment, and prioritizing vulnerable populations remain central to the objectives of primary health services.

## Graphical abstract

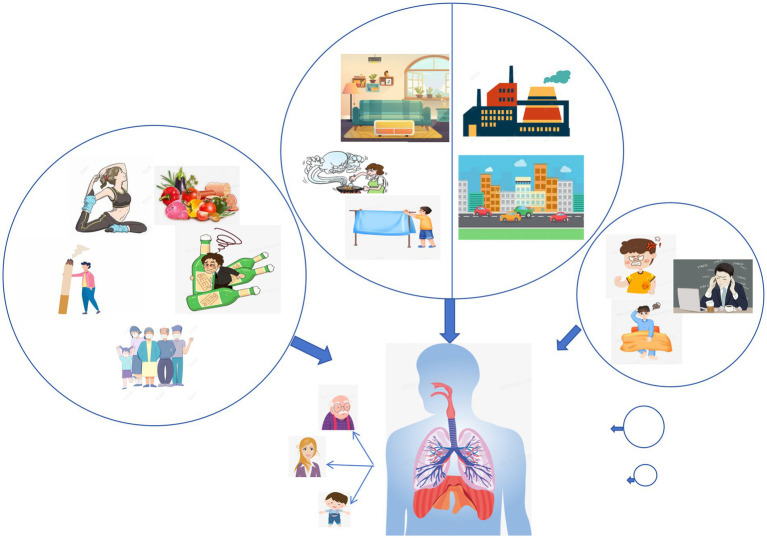

## 1 Introduction

Respiratory diseases encompass a range of conditions that impact the respiratory system. The high prevalence of these conditions poses a significant threat to both the physical and mental health of the population (1). In China, the Health Commission reports that respiratory diseases rank among the leading health concerns for residents, resulting in hundreds of thousands of deaths annually (2).

Research indicates that climate change is contributing to an increase in the frequency of extreme weather events, including elevated temperatures and heavy rainfall. These alterations in climate conditions adversely affect air quality and heighten the prevalence of respiratory diseases (1). Specifically, greenhouse gas emissions are closely associated with exacerbations of asthma and chronic obstructive pulmonary disease (COPD) (3). Additionally, air pollutants such as inhalable fine particulate matter (PM_2.5_), toxic gasses, and ozone have been shown to have significant correlations with the incidence of lung cancer, bronchitis, and other respiratory ailments. Environmental factors can influence the dysbiosis of the human airway organ microbiota (4) and may also impact genetic changes (5). Current domestic research primarily concentrates on coastal cities in the eastern and southeastern regions, where rising emissions from motor vehicles and industrial activities have resulted in severe air pollution (2). Moreover, lifestyle factors, including a lack of physical activity, an unbalanced diet, and smoking habits, indoor factors, including solid fuel combustion, cooking methods, and allergens from household pests, can adversely affect human health (6, 7).

While the aforementioned factors have been shown to independently influence the occurrence, progression, and even mortality associated with respiratory diseases in the population, recent drastic changes in global climate have led to unpredictable alterations in both the outdoor environment and personal living habits. In addition, regional factors, economic status, and other variables may also influence the incidence of respiratory diseases. Southwest China, characterized by its distinctive geographical location, relatively underdeveloped economic conditions, and limited medical resources, presents a particularly intriguing case for study. This uniqueness contributes to the novelty of our research. Consequently, we conducted a cross-sectional study in a southwestern region of China to examine living habits, indoor and outdoor environments, as well as personal and family medical histories. Our objective was to elucidate the relationship between these factors and the incidence of respiratory diseases. This study focuses on a distinct region, and to our knowledge, no similar research has been conducted in this area previously. The sample size is substantial, and the analysis is thorough. A nomogram was employed for predictive analysis of different respiratory diseases, and interaction effects were also examined. Additionally, we conducted data mining to identify key groups and principal influencing factors. However, in the cross-sectional study employed in this research, data are typically gathered through questionnaires without the inclusion of accompanying clinical measurements. This approach may result in recall bias and information bias. This research, on one hand, it offers essential data for the prevention and control of respiratory diseases within the region, while also developing and validating a nomogram to predict these diseases. This tool aids clinicians and community workers in identifying sensitive and high-risk populations. On the other hand, a comprehensive examination of the relationship between various dimensions and respiratory diseases across different subgroups will facilitate the development of more effective policies aimed at reducing the disease burden on residents and enhancing the overall quality of life for the population.

All investigations and analyses in this study strictly adhered to the STROBE guidelines for observational studies.

## 2 Materials and methods

### 2.1 Time and location

This study was conducted in a region of southwest China from February 2024 to May 2024. The region features a subtropical humid climate and encompasses both the Chengdu Plain and the mountainous terrains to the east. The distinctive landform and climate of the area contribute to the residents’ unique living habits, thereby enhancing the novelty and urgency of the research.

### 2.2 Sample size and the study population

According to the China Health Statistics Yearbook,^1^ the total prevalence of respiratory diseases in the population is *p* = 9.32%, *Q* = 1-*P*, *α* = 0.05, *Z*_α_ ≈ 2.00, *d* = 0.1P. The formula (8) is as follows:

$$N={Z_{\alpha }}^{2}PQ/d^{2}$$

Due to the presence of missing and biased information in the questionnaire utilized during the survey, the invalid sample rate was determined to be 15%. Consequently, the actual sample size was 4,579 individuals. In the survey, try to take into account the gender composition ratio, inclusion criteria for the personnel:

1. The study subjects were registered in the area and had lived in the local area for more than 5 years.
2. Clear consciousness, and be able to express their wishes correctly.
3. Subjects were informed and consented to the survey.

The opposite is ruled out.

In the design of the questionnaire, we ensured the removal of personal privacy information, including names, phone numbers, and identification numbers. The research team consistently adhered to the recommendations outlined in the Declaration of Helsinki during both the survey design and information processing stages.

### 2.3 Disease confirmation

We validated the classification of diseases in accordance with the International Statistical Classification of Diseases, Tenth Revision (ICD-10), focusing on the codes for Respiratory Diseases (J00-J99). The current health status of the individual under investigation must be confirmed by the individual themselves, their immediate family members, or their guardians. The diagnosis of cases is sourced from medical institutions at the township (community) level and above. For surveys conducted in the same area, we strive to collect diagnostic reports from the same hospital or from local hospitals of equivalent levels. This approach is intended to ensure the scientific rigor and accuracy of the diagnostic results while minimizing selection bias. Based on ICD-10 coding and consultations with several clinical experts holding senior professional titles, we classified the codes J00–J06, J18, and J20–J22 as acute respiratory diseases. We defined the codes J30–J32, J40–J47, and J60–J70 as chronic respiratory diseases, while the remaining codes were categorized as other respiratory diseases. In cases where participants presented with both acute and chronic respiratory diseases, we concentrated on analyzing the types of morbidity reported by the participants during the survey period, guided by the insights of clinical experts. During our on-site investigation, we will concentrate on inquiring about and verifying the existence of written or electronic inspection reports for participants who have a history of acute and chronic diseases.

### 2.4 Investigation method and content

We employed a multi-stage stratified sampling method to randomly select three areas from a total of six jurisdictions within the region and conducted epidemiological surveys among the residents of the selected areas. The three regions we selected encompass over 68% of the population within the entire survey area. With the support of the local CDC and community workers, we established several monitoring points in these high-traffic areas. Each monitoring point effectively covers the entire region, allowing the samples we collected to adequately represent the overall population of southwest China. The questionnaire was developed based on the Community Respiratory Health Cohort in Europe, the Chinese Urban Cancer Screening Questionnaire, and prior studies conducted in China (3, 9, 10). The content of the questionnaire was reviewed and revised by two experts with senior professional titles. In our research, we aim to reduce the time frame for problem setting to within 5 years. Most problems exist objectively or have been defined to minimize subjective impact. Prior to the survey, investigators received unified professional training, and all questionnaire data were collected through face-to-face interviews to minimize recall bias.

### 2.5 Definition of influencing factors

We categorize the influencing factors into five dimensions: personal circumstances, environmental factors, individual health status, family medical history, and additional variables. In our analysis, we synthesize personal medical history, familial health history, and other relevant factors. We categorize age into three groups: “0–14 years,” “15–64 years,” and “>65 years,” in accordance with the international age structure. Subjects who quit smoking for more than 1 year were defined as ever-smokers. Drinking was defined as the consumption of more than one drink (8 g of alcohol) per day for women and more than two drinks per day for men. Engaging in moderate activity, which includes aerobic exercise, strength training, mobility and flexibility exercises, and balance training for 40 min or more each day, along with high-intensity activity, is regarded as a healthy amount of exercise. According to the recommendations of the World Health Organization (WHO), a daily salt intake exceeding 5 g (approximately one teaspoon) is classified as high salt consumption. February 2024 to March 2024 is defined as the cold season, while April 2024 to May 2024 is designated as the warm season. If the subject has experienced an acute upper respiratory tract infection (such as a cold, rhinitis, or pharyngitis/laryngitis), influenza, or pneumonia within the past 2 weeks, they are defined as currently diagnosed with a respiratory disease. An acute lower respiratory tract infection—such as acute bronchitis, a new coronavirus infection, bronchopneumonia, or bronchiolitis—occurring within the past 4 weeks is diagnosed as respiratory disease. Subjects were classified as having a history of respiratory diseases if they had experienced asthma, bronchitis, COVID-19, or other severe respiratory conditions within the past year. Environmental factors can be divided into two types: indoor and outdoor conditions. If the illumination rate, defined as the ratio of window area to floor area, of each habitable room exceeds 12.5% (or 1/8), it is considered to provide good lighting. For further details, please refer to the relevant references (11). Based on previous studies and indoor ventilation building standards (6, 7, 12), we define daily ventilation as mechanical ventilation with windows open for more than 8 h a day or the use of air purification equipment (including ventilators, exhaust vents, etc.) for more than 2 h a day. Chronic expectoration, occupational dust/toxic gas exposure history was defined in Fang et al. (3). In the absence of a specific definition for house dust mites, we employed a combination of participant observation methods (13) and a diagnosis of dust mite allergy provided by a clinical expert to determine the presence of dust mites within the home. The remaining influencing factors did not need to be quantified, and the results were all yes/no responses.

### 2.6 Statistical analysis

Data processing was conducted using Microsoft Office 2007, while all analyses were performed using SPSS version 26.0 and R version 4.3.0. We tabulated the resident characteristics by the presence or absence of respiratory disease. Baseline characteristics were expressed as numbers and percentages for categorical variables and compared using the chi-square test or Fisher test (as appropriate). We divided the dataset into a training set and a test set. We then performed lasso-logistic regression on all factors within both the training and test sets, adjusted for collinearity among variables, and screened for influencing factors. Plot the receiver operating characteristic (ROC) curves for both the training set and validation set, and calculate the area under the curve (AUC). Additionally, generate the Hosmer-Lemeshow (HL) curve, clinical decision curve (DCA), and the model’s confusion matrix to assess model performance and calibration, establish a nomogram prediction model to evaluate the impact of each factor on the disease and its predicted probability.

In this study, certain variables are multi-categorical, rendering additive interaction inapplicable. Consequently, this article consistently employs the ‘interactionR’ package to perform multiplicative interaction model analysis (14). In the multiplicative interaction model, we utilize gender and age group as stratification variables (independent variables) for conducting stratified analyses. The filtered factors serve as interaction variables (analytic variables) for subgroup interaction analysis. Additionally, we control for co-factors such as gender, age, height, weight, income, and occupation, allowing us to analyze the impact of each factor on individuals across different genders and age groups. *p* < 0.05 was considered as statistical significance.

## 3 Results

### 3.1 Basic information

#### 3.1.1 Demographic characteristics

The study initially aimed to include 4,579 participants while excluding invalid sample. Ultimately, 4,507 respondents were incorporated into the analysis, resulting in a questionnaire response rate of 98.43% and a crude prevalence rate of 21.21%. In the sample, 22.57% of men and 19.99% of women were sicken. The older adult group (> 65 years) exhibited the highest prevalence at 30.28%. Additionally, production and transportation equipment operators, along with related personnel, demonstrated the highest prevalence rate of 33.96%. The total number of patients with acute respiratory tract disease is 782, while the number of patients with chronic respiratory tract disease is 238. See Table 1 for the rest.

**Table 1:** 2024.2–2024.5 basic demographic information and respiratory diseases of permanent residents in a certain area.

Variables	Not sick (*n* = 3,551)	Sicken (*n* = 956)	Sicken			Total (*n* = 4,507)
			Acute (*n* = 782)	Chronic (*n* = 238)	Other (*n* = 71)	
Sex, *n*(%)						
Male	1,654 (46.58)	482 (50.42)	362 (46.29)	150 (63.03)	47 (66.20)	2,136 (47.39)
Female	1897 (53.42)	474 (49.58)	420 (53.71)	88 (36.97)	24 (33.80)	2,371 (52.61)
*p-*value	0.035		0.497	<0.0001	0.001	
Age group, *n*(%)						
0–14	1,020 (28.72)	164 (17.15)	159 (20.33)	11 (4.62)	2 (2.82)	1,184 (26.27)
15–64	1716 (48.32)	438 (45.82)	373 (47.70)	88 (36.97)	42 (59.15)	2,154 (47.79)
>65	815 (22.95)	354 (37.03)	250 (31.97)	139 (58.40)	27 (38.03)	1,169 (25.94)
*P-*value	<0.0001		<0.0001	<0.0001	<0.0001	
Marriage, *n*(%)						
Single	1,250 (35.20)	247 (25.84)	236 (30.18)	22 (9.24)	6 (8.45)	1,497 (33.21)
Married	2,143 (60.35)	631 (66.00)	496 (63.43)	182 (76.47)	58 (81.69)	2,774 (61.55)
Divorce or separation	71 (2.00)	25 (2.62)	17 (2.17)	7 (2.94)	3 (4.23)	96 (2.13)
Widow	87 (2.45)	53 (5.54)	33 (4.22)	27 (11.34)	4 (5.63)	140 (3.11)
*P-*value	<0.0001		0.073	<0.0001	<0.0001	
Income (RMB, /month), *n*(%)						
0–2,999	2,453 (69.08)	618 (64.64)	502 (64.19)	172 (72.27)	47 (66.20)	3,071 (68.14)
3,000–7,999	917 (25.82)	288 (30.13)	234 (29.92)	62 (26.05)	21 (29.58)	1,205 (26.74)
8,000–14,999	106 (2.99)	25 (2.62)	21 (2.69)	3 (1.26)	3 (4.23)	131 (2.91)
15,000–29,999	30 (0.84)	6 (0.63)	6 (0.77)	1 (0.42)	0 (0.00)	36 (0.80)
>30,000	45 (1.27)	19 (1.99)	19 (2.43)	0 (0.00)	0 (0.00)	64 (1.42)
*P-*value	0.027		0.013	0.314	0.820	
Medical Insurance, *n*(%)						
Self-paying	418 (11.77)	103 (10.77)	86 (11.00)	25 (10.50)	7 (9.86)	521 (11.56)
Basic medical insurance for urban workers	970 (27.32)	317 (33.16)	264 (33.76)	80 (33.61)	23 (32.39)	1,287 (28.56)
Medical insurance for both urban and rural residents	2019 (56.86)	506 (52.93)	411 (52.56)	129 (54.20)	32 (45.07)	2,525 (56.02)
Commercial health insurance	34 (0.96)	10 (1.05)	6 (0.77)	1 (0.42)	3 (4.23)	44 (0.98)
Other	110 (3.10)	20 (2.09)	15 (1.92)	3 (1.26)	6 (8.45)	130 (2.88)
*P-*value	0.006		0.005	0.220	0.001	
Education, *n*(%)						
Primary school and below	945 (26.61)	298 (31.17)	240 (30.69)	89 (37.39)	18 (25.35)	1,243 (27.58)
Junior middle school	1,113 (31.34)	239 (25.00)	186 (23.79)	61 (25.63)	25 (35.21)	1,352 (30.00)
Senior middle school	407 (11.46)	119 (12.45)	91 (11.64)	36 (15.13)	7 (9.86)	526 (11.67)
Junior college education	672 (18.92)	165 (17.26)	145 (18.54)	31 (13.03)	14 (19.72)	837 (18.57)
Undergraduate course	363 (10.22)	115 (12.03)	103 (13.17)	17 (7.14)	7 (9.86)	478 (10.61)
Graduate student or above	51 (1.44)	20 (2.09)	17 (2.17)	4 (1.68)	0 (0.00)	71 (1.58)
*P-*value	<0.0001		<0.0001	0.001	0.819	
Nationality, *n*(%)						
Han ethnic	3,475 (97.86)	923 (96.55)	755 (96.55)	232 (97.48)	69 (97.18)	4,398 (97.58)
Uygur ethnic	11 (0.31)	11 (1.15)	9 (1.15)	2 (0.84)	0 (0.00)	22 (0.49)
Kazak ethnic	23 (0.65)	15 (1.57)	14 (1.79)	3 (1.26)	0 (0.00)	38 (0.84)
Yi ethnic	14 (0.39)	1 (0.10)	0 (0.00)	0 (0.00)	1 (1.41)	15 (0.33)
Hui ethnic	10 (0.28)	4 (0.42)	2 (0.26)	1 (0.42)	1 (1.41)	14 (0.31)
Other ethnic	18 (0.51)	2 (0.21)	2 (0.26)	0 (0.00)	0 (0.00)	20 (0.44)
*P-*value	<0.0001		<0.0001	0.578	0.239	
Occupation, *n*(%)						
Student	987 (27.79)	155 (16.21)	149 (19.05)	9 (3.78)	2 (2.82)	1,142 (25.34)
Responsible person of each unit	94 (2.65)	44 (4.60)	40 (5.12)	8 (3.36)	5 (7.04)	138 (3.06)
Professionals	537 (15.12)	172 (17.99)	148 (18.93)	42 (17.65)	15 (21.13)	709 (15.73)
Staff members	74 (2.08)	26 (2.72)	21 (2.69)	5 (2.10)	2 (2.82)	100 (2.22)
Business and service industry personnel	177 (4.98)	55 (5.75)	46 (5.88)	10 (4.20)	2 (2.82)	232 (5.15)
Agriculture/forestry/fishing. et al. personnel	793 (22.33)	280 (29.29)	210 (26.85)	101 (42.44)	15 (21.13)	1,073 (23.81)
Operators of the production and transportation equipment	35 (0.99)	18 (1.88)	10 (1.28)	9 (3.78)	2 (2.82)	53 (1.18)
Soldier	6 (0.17)	1 (0.10)	1 (0.13)	0 (0.00)	0 (0.00)	7 (0.16)
Others and the retirees	848 (23.88)	205 (21.44)	157 (20.08)	54 (22.69)	28 (39.44)	1,053 (23.36)
*P-*value	<0.0001		<0.0001	<0.0001	<0.0001	

#### 3.1.2 Living habits

Supplementary Table S1 show that compared to healthy individuals, various types of patients exhibit distinct degrees of significant differences in their personal living habits. Among the total population, as well as those suffering from acute and chronic respiratory diseases, current smoking, daily exposure to second-hand smoke, heavy alcohol consumption, excessive dietary habits, and other detrimental lifestyle choices constitute the largest contributing factors. The majority of healthy individuals maintain good lifestyle habits, which include regular exercise, a balanced diet and so on.

### 3.2 Indoor and outdoor environmental factors

#### 3.2.1 Indoor environment

Supplementary Table S2 result show: indoor environments often contain significant levels of oil fumes, particularly in settings where a large volume of cooking products is utilized, and the presence of long-haired pets has been linked to an increased prevalence of disease within the population. Implementing regular indoor ventilation, ensuring adequate lighting, conducting frequent cleaning and disinfection, and utilizing clean fuels are associated with low disease rates. There is no statistically significant difference in disease among people who use floor heating and electric stoves for heating in winter, use electricity and coal for cooking.

#### 3.2.2 Outdoor factors

Supplementary Table S3 show higher prevalence in the cold season (24.64%) than in the warm season (17.83%). People who have a history of exposure to occupational dust/toxic gasses and toxic metals have higher prevalence rates than those who do not have the above conditions.

### 3.3 Personal history of the disease, family history, and other factors

Supplementary Tables S4–S6 show that individuals with a personal history of respiratory disease, as well as those with a family history of such conditions, exhibit a higher prevalence compared to those without any history of respiratory disease. Individuals who frequently cough and produce phlegm, experience mood disturbances, and suffer from poor sleep quality are significantly associated with respiratory diseases.

### 3.4 Lasso regression

**Figure 1:**
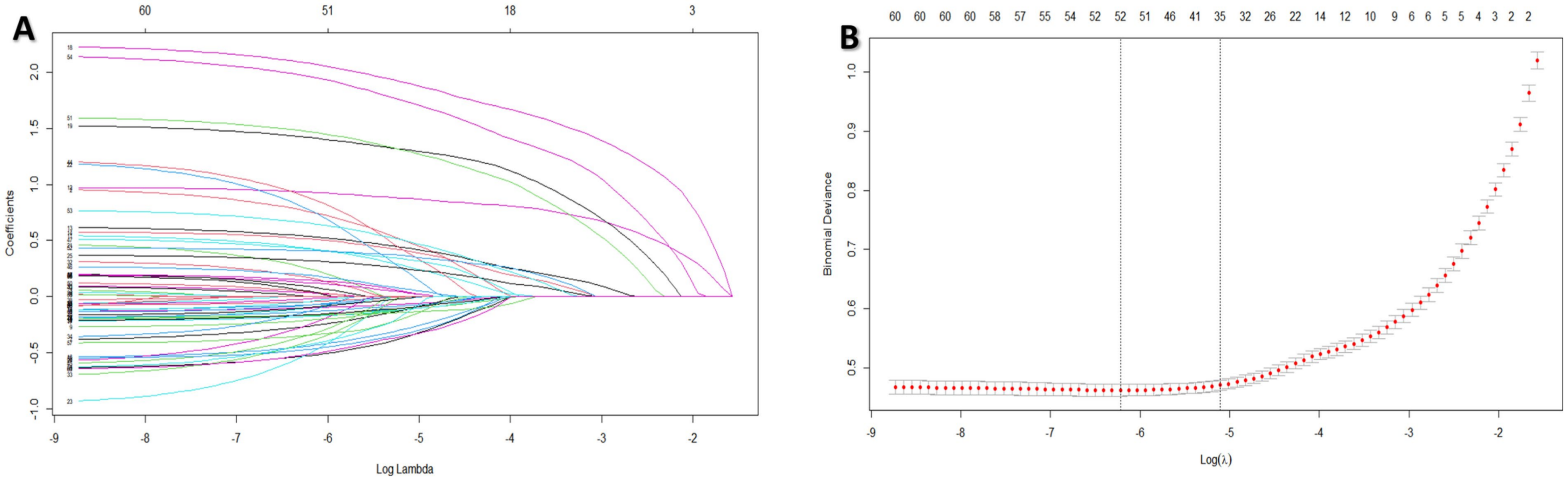
2024.2–2024.5 factors influencing respiratory diseases of permanent residents in a certain area were screened through the process and results by lasso regression.

We randomly divided the dataset into a training set and a validation set in a 7:3 ratio. The penalty coefficient in lasso regression is utilized in both the training and test sets to mitigate the influence of correlated features. This approach stabilizes the model, enhances its generalization capabilities, and effectively addresses multicollinearity among variables. We incorporate the L1 regularization term into the loss function depicted in Figure 1A. This addition causes the coefficients of certain features to become zero, thereby facilitating automatic feature selection. The initial set of 60 factors was reduced to 35 variables (Figure 1B), resulting in a model that exhibits optimal fitting performance. The selected variables are presented in Supplementary Table S7.

### 3.5 Logistic stepwise regression

**Figure 2:**
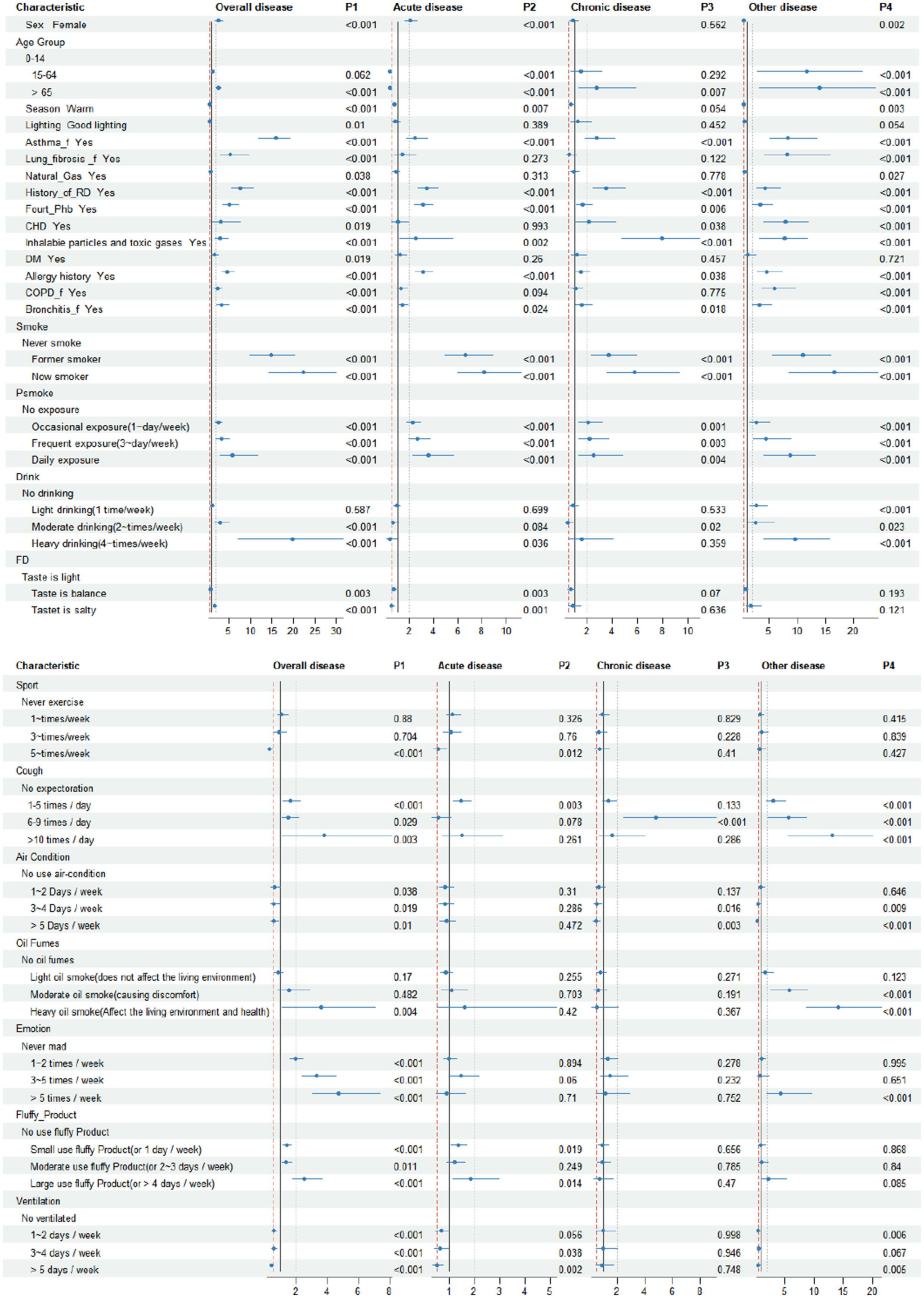
2024.2–2024.5 Results of factors influencing respiratory diseases in a permanent resident screened by logistic regression. Psmoke, passive smoke; FD, food taste; ventilation, natural ventilation or mechanical ventilation; Oil Fumes, cooking oil fumes; air condition, use air condition in summer; DM, dust mite. Fourt Phb, whether there is hospitalized for severe respiratory diseases such as pneumonia and bronchitis before the age of 14 years; CHD, coronary heart disease. X_f, family history of X. Emotion, the survey period was within 5 years.

We subsequently employed the logistic stepwise regression method to evaluate the 35 variables identified through lasso regression, ultimately selecting 26 factors for further analysis. The findings are presented in Figure 2.

### 3.6 Prediction model

#### 3.6.1 Draw the normograph

**Figure 3:**
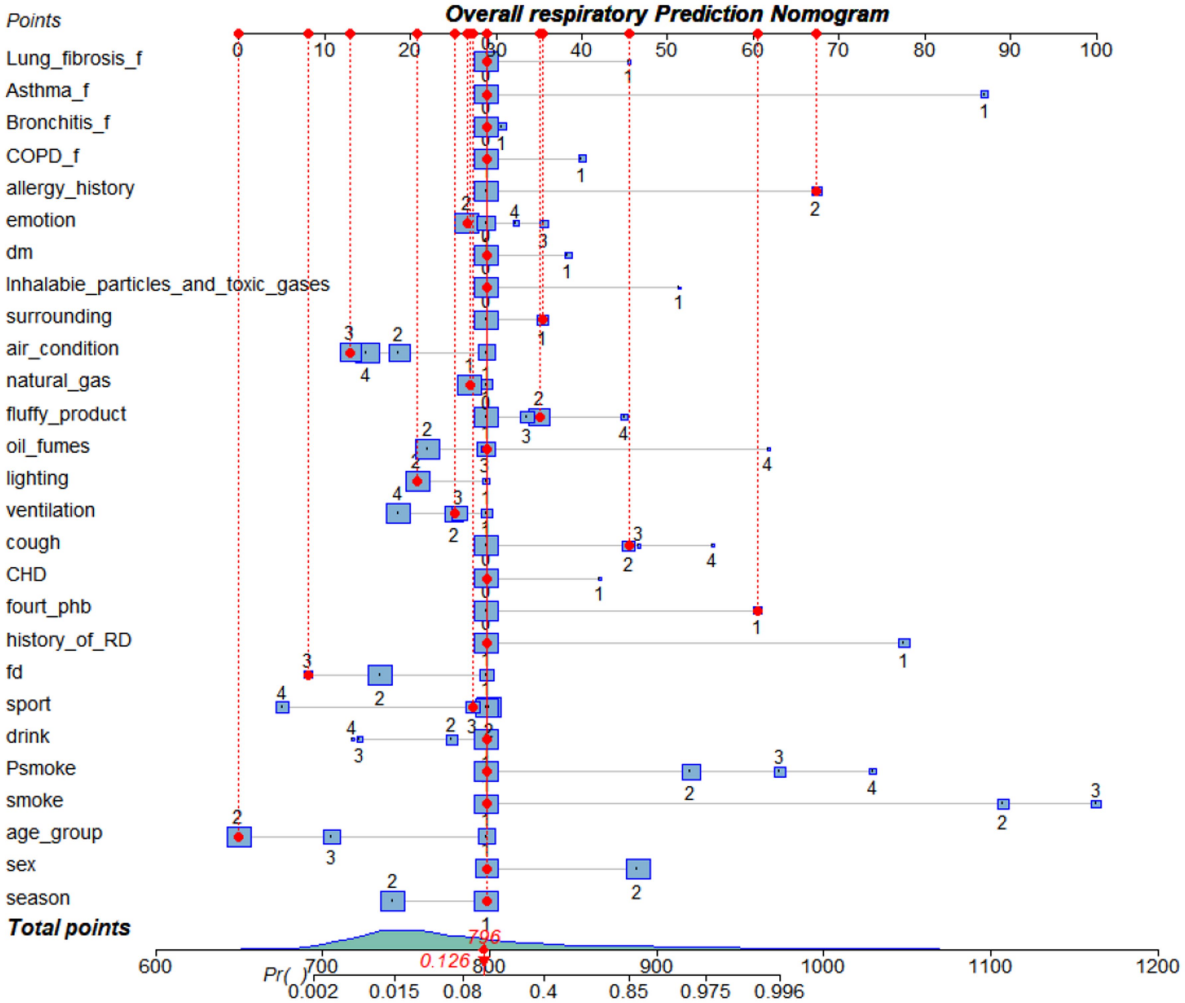
Nomogram of predicted overall respiratory disease risk established after lasso-logistic regression analysis. Fourt Phb, whether there is hospitalized for severe respiratory diseases such as pneumonia and bronchitis before the age of 14 years. Psmoke, passive smoke; FD, food taste; ventilation: natural ventilation or mechanical ventilation; oil fumes, cooking oil fumes; air condition, use air condition in summer; DM, dust mite. Fourt Phb, whether there is hospitalized for severe respiratory diseases such as pneumonia and bronchitis before the age of 14 years; CHD, coronary heart disease. X_f, family history of X.

We retain the logistic stepwise regression model results for personal factors (such as sex, age group, smoke, Psmoke, drink, sport, fd), we retain the environment factors (such as ventilation, lighting, natural gas, fluffy product, oil fumes, air condition, dm, season, inhalable particles and toxic gasses), we retain the other factors (such as four phb, allergy history, expectation, history of RD, CHD, Bronchitis f, CODP f, lung fibrosis f, asthma f, emotion to draw the nomogram). Figure 3 is a nomogram of the overall respiratory disease population. Supplementary Figures S1, S3 present nomograms for acute respiratory tract diseases and chronic respiratory diseases, respectively.

#### 3.6.2 Performance evaluation of the prediction model

**Figure 4:**
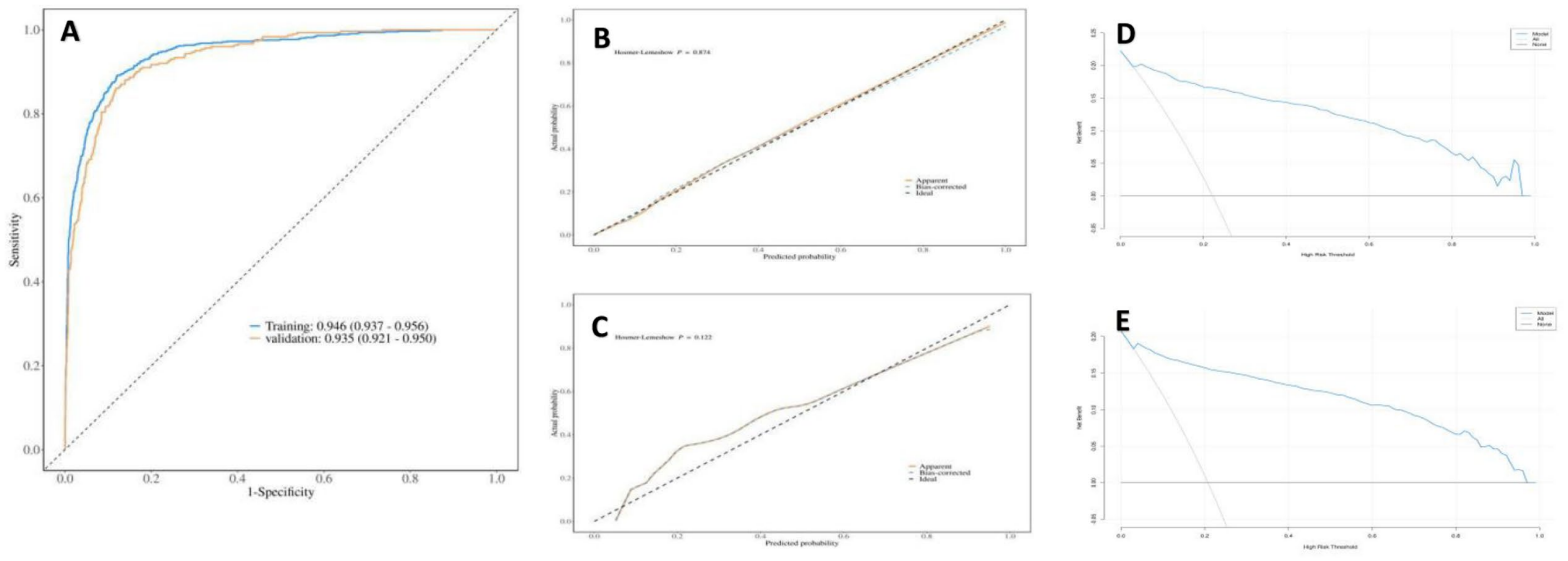
Receiver operating characteristic (ROC) curve and AUC of the area under the curve of the training set and validation set in the nomogram for predicting overall respiratory diseases, calibration curve H-L and clinical decision curve DCA.

Figure 4A show that: in the overall respiratory prediction model, the area under the ROC curve was 0.946 (95% CI: 0.937, 0.956) for the training set and 0.935 (95% CI: 0.921, 0.950) for the validation set, indicating superior performance. The calibration curve demonstrated strong agreement between the training and validation data sets (Figures 4B,C). The training set DCA indicates that when an individual’s threshold probability ranges from 5 to 95%, and the validation set DCA shows a threshold probability range of 6 to 93%, the application of this model for predicting respiratory diseases demonstrates the greatest clinical benefit (Figures 4D,E). The results of the model’s confusion matrix indicate that the accuracy, sensitivity, and specificity of the model are all high (Table 2). Supplementary Figures S2, S4, along with Supplementary Tables S8, S9 demonstrate that the prediction models for both acute and chronic respiratory disease exhibit superior performance.

**Table 2:** Results of the confusion matrix for the nomogram for predicting overall respiratory diseases.

Data	AUC (95%CI)	Accuracy (95%CI)	Sensitivity (95%CI)	Specificity (95%CI)	PPV (95%CI)	NPV (95%CI)	cut off
Train	0.946 (0.937–0.956)	0.903 (0.892–0.913)	0.914 (0.903–0.925)	0.858 (0.831–0.885)	0.951 (0.943–0.959)	0.724 (0.693–0.756)	0.246
Test	0.935 (0.921–0.950)	0.905 (0.888–0.920)	0.924 (0.908–0.940)	0.837 (0.796–0.879)	0.942 (0.929–0.955)	0.759 (0.713–0.805)	0.246

### 3.7 Interactive analysis

We used gender and age group as stratification variables (independent variables). Based on the results of this study, we identified meaningful factors from both personal and environmental categories, which were utilized as interaction variables for conducting subgroup interaction analysis and sensitivity analysis. Based on previous studies (15–18) and the findings of this research, we controlled for covariates including income, occupation, type of medical insurance, education, ethnicity, marital status, family history, disease history and other factors.

#### 3.7.1 Living habits

The interaction among gender, age group, and smoking status reveals significant findings. Among individuals with acute respiratory illnesses, women and older adults who are current smokers are at the highest risk. Additionally, adolescents and the older adult exposed to secondhand smoke face a greater risk compared to young adults and middle-aged individuals (age 15–64). In the context of chronic respiratory diseases, tobacco exposure poses a more severe threat to the older adult, while the frequency of weekly exercise in women is notably correlated with a reduced risk of disease. Moreover, the frequency of exposure to tobacco is positively associated with the risk of disease, as indicated by an increased odds ratio (OR). Furthermore, there exists an interaction between gender and alcohol consumption; women who consume alcohol are at a higher risk of developing alcohol-related diseases than men, and an increase in weekly alcohol intake correlates with an elevated risk. See details in Tables 3, 4 and Supplementary Tables S10, S11.

**Table 3:** Subgroup interaction sheet between gender and lifestyle factors.

Characteristic	Overall disease		Acute disease		Chronic disease		Other disease	
	OR(95%CI)	*P* for interaction	OR(95%CI)	*P* for interaction	OR(95%CI)	*P* for interaction	OR(95%CI)	*P* for interaction
Smoke		<0.001		<0.001		0.468		0.107
Sex.1_smoke.1	Ref		Ref		Ref		Ref	
Sex.2_smoke.1	1.41(1.09,1.81)***		1.14(0.87,1.48)		1.36(0.72,2.55)		1.90(0.51,7.09)	
Sex.1_smoke.2	8.83(6.55,11.90)***		3.73(2.74,5.08)***		9.88(5.38,18.14)***		16.10(4.68,55.38)***	
Sex.2_smoke.2	17.13(8.48,29.83)***		11.75(8.18,16.88)***		8.67(4.41,17.07)***		8.23(1.92,35.23)**	
Sex.1_smoke.3	10.12(7.47,13.72)***		3.32(2.40,4.59)***		12.38(6.72,22.84)***		16.26(4.66,56.67)***	
Sex.2_smoke.3	20.59(9.17,31.86)***		41.17(24.93,67.97)***		15.73(7.87,31.45)***		28.13(7.51,105.30)***	
Psmoke		0.143		0.041		0.262		0.078
Sex.1_Psmoke.1	Ref		Ref		Ref		Ref	
Sex.2_Psmoke.1	1.19(0.89,1.58)		2.21(1.59,3.08)***		1.91(1.02,3.56)*		0.94(0.33,2.65)	
Sex.1_Psmoke.2	4.57(3.46,6.02)***		3.24(2.37,4.45)***		4.31(2.54,7.31)***		1.59(0.72,3.52)	
Sex.2_Psmoke.2	3.74(2.84,4.93)***		6.31(4.58,8.68)***		4.05(2.27,7.23)***		2.63(1.12,6.16)*	
Sex.1_Psmoke.3	8.40(6.05,11.66)***		3.94(2.70,5.75)***		5.06(2.83,9.03)***		1.83(0.74,4.52)	
Sex.2_Psmoke.3	10.20(7.11,14.64)***		12.40(8.27,18.61)***		4.98(2.47,10.03)***		2.50(0.83,7.57)	
Sex.1_Psmoke.4	15.50(9.84,24.40)***		4.50(2.74,7.40)***		6.56(3.35,12.83)***		3.9414(1.56,9.97)*	
Sex.2_Psmoke.4	18.71(10.51,33.30)***		22.63(12.04,42.53)***		5.84(2.32,14.70)***		NA	
Drink		<0.001		0.165		0.683		0.318
Sex.1_drink.1	Ref		Ref		Ref		Ref	
Sex.2_drink.1	1.10(0.90,1.35)		2.13(1.67,2.71)***		1.03(0.70,1.5)		1.03(0.51,2.07)	
Sex.1_drink.2	2.05(1.60,2.64)***		0.80(0.60,1.06)		1.02(0.68,1.53)		1.29(0.65,2.57)	
Sex.2_drink.2	1.81(1.29,2.56)***		2.67(1.81,3.94)***		1.24(0.66,2.33)		0.98(0.28,3.48)	
Sex.1_drink.3	4.82(3.44,6.77)***		0.61(0.41,0.91)*		0.63(0.35,1.11)		0.65(0.23,1.86)	
Sex.2_drink.3	16.49(8.02,33.88)***		0.85(0.27,2.63)		1.00(0.21,4.77)		3.88(0.76,19.75)	
Sex.1_drink.4	14.65(7.51,28.57)***		0.38(0.17,0.84)*		1.04(0.44,2.46)		3.00(0.98,9.14)	
Sex.2_drink.4	22.52(10.92,45.70)***		0.42(0.04,3.97)		3.54(0.52,24.27)		7.07(0.68,73.14)	
Sport		0.006		0.018		0.017		0.844
Sex.1_sport.1	Ref		Ref		Ref		Ref	
Sex.2_sport.1	0.80(0.63,1.01)		2.52(1.86,3.43)***		1.08(0.69,1.69)		1.30(0.56,3.01)	
Sex.1_sport.2	0.79(0.62,1.01)		0.98(0.73,1.31)		0.69(0.46,1.03)		1.13(0.56,2.30)	
Sex.2_sport.2	0.67(0.53,0.85)***		2.16(1.60,2.93)		0.75(0.46,1.20)		0.97(0.39,2.41)	
Sex.1_sport.3	0.74(0.54,1.03)		1.14(0.77,1.69)		0.47(0.24,0.916)*		1.64(0.67,3.98)	
Sex.2_sport.3	0.51(0.36,0.72)***		1.75(1.14,2.69)*		0.85(0.41,1.78)		1.89(0.59,6.11)	
Sex.1_sport.4	0.32(0.21,0.49)***		0.37(0.21,0.63)***		0.59(0.41,0.82)**		0.87(0.28,2.70)	
Sex.2_sport.4	0.23(0.13,0.48)***		0.29(0.14,0.58)***		0.44(0.23,0.67)***		1.61(0.34,7.59)	

Sex1: male; sex2: female; smoke 1, 2, 3: never smoke former smoker, now smoker; Psmoke 1, 2, 3, 4: no exposure, 1 ~ 2 days/week, 3 ~ 4 days/week, daily exposure; drink 1, 2, 3, 4: no drinking, 1 time/week, 2 ~ 3 times/week, >4 times/week; sport 1, 2, 3, 4: never exercise, 1 ~ 2 times/week, 3 ~ 4 times/week, >5 times/week. ***p < 0.0001, **p < 0.001, *p < 0.05.

**Table 4:** Age group, subgroup interaction sheet between lifestyle factors.

Characteristic	Overall disease		Acute disease		Chronic disease		Other disease	
	OR(95%CI)	*P* for interaction	OR(95%CI)	*P* for interaction	OR(95%CI)	*P* for interaction	OR(95%CI)	*P* for interaction
Smoke		<0.001		<0.001		<0.001		0.045
Age_group.1_smoke.1	Ref		Ref		Ref		Ref	
Age_group.2_smoke.1	0.23(0.14,0.37)***		0.28(0.21,0.38)***		0.36(0.13,0.98)*		4.92(0.60,40.22)	
Age_group.3_smoke.1	0.62(0.39,1.00)*		0.77(0.57,1.05)		4.15(2.05,8.38)***		5.67(0.63,51.14)	
Age_group.1_smoke.2	3.05(1.28,7.26)**		2.11(0.86,5.14)		NA		31.28(1.84,531.30)*	
Age_group.2_smoke.2	4.54(2.72,6.73)***		3.97(2.96,5.34)***		7.01(3.45,14.24)***		22.85(2.85,182.97)*	
Age_group.3_smoke.2	3.86(2.33,6.40)***		2.64(1.86,3.75)***		19.92(9.95,39.90)***		55.47(7.09,433.83)***	
Age_group.1_smoke.3	4.60(3.42,6.01)***		1.97(0.51,7.60)		NA		NA	
Age_group.2_smoke.3	5.23(3.26,8.40)***		3.91(2.84,5.37)***		11.35(5.64,22.88)***		65.04(8.50,497.85)***	
Age_group.3_smoke.3	6.40(3.77,10.86)***		3.68(2.55,5.31)***		23.10(11.40,46.79)***		29.63(3.54,247.65)*	
Psmoke		<0.001		0.034		0.004		0.56
Age_group.1_Psmoke.1	Ref		Ref		Ref		Ref	
Age_group.2_Psmoke.1	1.24(0.74,2.08)		0.58(0.39,0.86)**		1.14(0.41,3.21)		3.15(0.39,25.64)	
Age_group.3_Psmoke.1	1.98(1.15,3.42)*		0.83(0.55,1.25)		2.75(1.02,7.46)*		3.07(0.35,26.60)	
Age_group.1_Psmoke.2	3.68(2.49,5.44)***		3.89(2.60,5.81)***		1.92(0.51,7.26)		NA	
Age_group.2_Psmoke.2	4.35(2.63,7.19)***		1.90(1.33,2.72)***		3.21(1.22,8.46)*		6.35(0.80,50.13)	
Age_group.3_Psmoke.2	6.51(3.80,11.16)***		2.11(1.42,3.14)***		9.83(3.78,25.58)***		6.30(0.77,51.78)	
Age_group.1_Psmoke.3	11.07(6.48,18.92)***		11.75(6.81,20.28)***		3.68(0.69,19.74)		NA	
Age_group.2_Psmoke.3	7.15(4.16,12.27)***		2.48(1.61,3.80)***		2.06(0.69,6.17)		6.71(0.79,56.89)	
Age_group.3_Psmoke.3	22.08(11.95,40.79)***		2.76(1.67,4.57)***		16.92(6.16,46.46)***		6.43(0.71,58.47)	
Age_group.1_Psmoke.4	14.62(4.83,44.28)***		10.07(3.14,32.33)***		NA		27.53(1.37,553.65)*	
Age_group.2_Psmoke.4	17.44(9.38,32.44)***		3.65(2.13,6.23)***		9.07(3.16,26.03)***		7.55(0.81,70.22)	
Age_group.3_Psmoke.4	22.88(10.61,43.95)***		4.55(2.22,9.33)***		8.30(2.54,27.12)***		10.63(1.05,107.36)*	
Drink		0.148		0.573		0.283		0.171
Age_group.1_drink.1	Ref		Ref		Ref		Ref	
Age_group.2_drink.1	0.70(0.45,1.09)		0.45(0.35,0.59)***		1.40(0.66,2.98)		5.77(0.74,44.65)	
Age_group.3_drink.1	1.10(0.68,1.76)		0.56(0.41,0.75)***		4.92(2.36,10.27)***		7.46(0.94,59.24)	
Age_group.1_drink.2	0.92(0.38,2.20)		0.41(0.14,1.23)		4.18(0.84,20.80)		NA	
Age_group.2_drink.2	1.23(0.77,1.96)		0.38(0.27,0.53)***		1.60(0.72,3.55)		8.77(1.10,70.18)*	
Age_group.3_drink.2	2.30(1.40,3.80)**		0.45(0.31,0.65)		3.65(1.65,8.04)**		5.18(0.60,45.11)	
Age_group.1_drink.3	NA		1.29(0.19,8.62)		NA		NA	
Age_group.2_drink.3	3.77(2.21,6.42)***		0.21(0.12,0.35)***		0.49(0.14,1.68)		3.81(0.37,39.53)	
Age_group.3_drink.3	6.61(3.58,12.20)***		0.26(0.15,0.46)***		3.06(1.23,7.63)*		6.85(0.70,66.97)	
Age_group.1_drink.4	NA		NA		NA		NA	
Age_group.2_drink.4	15.98(6.69,36.62)***		0.12(0.04,0.35)***		2.32(0.67,8.03)		15.29(1.41,165.74)*	
Age_group.3_drink.4	17.82(7.01,45.28)***		0.22(0.07,0.65)**		4.42(1.21,16.14)*		18.41(1.48,229.57)*	
Sport		0.344		0.392		0.227		0.235
Age_group.1_sport.1	Ref		Ref		Ref		Ref	
Age_group.2_sport.1	1.23(0.77,1.97)		0.44(0.30,0.65)***		1.32(0.45,3.90)		1.74(0.21,14.17)	
Age_group.3_sport.1	2.23(1.36,3.63)**		0.47(0.32,0.71)***		4.29(1.49,12.33)*		2.43(0.30,19.56)	
Age_group.1_sport.2	0.90(0.62,1.32)		0.82(0.55,1.24)		1.14(0.31,4.17)		NA	
Age_group.2_sport.2	0.99(0.62,1.58)		0.38(0.26,0.55)***		0.99(0.34,2.95)		2.45(0.31,19.34)	
Age_group.3_sport.2	1.80(1.09,2.98)*		0.54(0.35,0.83)**		3.92(1.33,11.55)*		1.41(0.15,12.79)	
Age_group.1_sport.3	0.80(0.49,1.31)		0.82(0.49,1.37)		NA		NA	
Age_group.2_sport.3	0.82(0.48,1.40)		0.32(0.19,0.52)***		1.26(0.38,4.19)		2.71(0.31,23.90)	
Age_group.3_sport.3	1.47(0.82,2.66)		0.45(0.26,0.80)***		2.30(0.68,7.78)		4.95(0.56,44.09)	
Age_group.1_sport.4	0.20(0.08,0.47)***		0.21(0.09,0.51)***		0.67(0.07,6.18)		2.99(0.18,49.17)	
Age_group.2_sport.4	0.64(0.35,1.14)		0.24(0.13,0.43)***		1.23(0.35,4.33)		2.18(0.23,20.79)	
Age_group.3_sport.4	0.68(0.34,1.34)		0.22(0.11,0.44)***		1.58(0.42,6.02)		0.92(0.05,15.62)	

Age group 1, 2, 3: “0–14,” “15–64,” “65~”; smoke 1, 2, 3: never smoke, former smoker, now smoker; Psmoke 1, 2, 3, 4: no exposure, 1 ~ 2 days/week, 3 ~ 4 days/week, daily exposure; drink 1, 2, 3, 4: no drinking, 1 time/week, 2 ~ 3 times/week, >4 times/week; sport 1, 2, 3, 4: never exercise, 1 ~ 2 times/week, 3 ~ 4 times/week, >5 times/week. ***P < 0.0001, **P < 0.001, *P < 0.05.

#### 3.7.2 Environmental factors

Tables 5, 6 and Supplementary Tables S12, S13 illustrate the interactions between age groups, gender, and ventilation conditions in indoor environments. Among acute respiratory diseases, women and the older adult demonstrate greater susceptibility to the adverse effects of kitchen smoke, with the older adult appearing to be particularly sensitive to house dust mites and air conditioning. In the context of chronic respiratory diseases, men show a higher susceptibility to kitchen smoke. The frequency of mechanical ventilation is inversely related to disease risk across all subgroups. Notably, the association between ventilation and reduced disease risk is more pronounced in women compared to the non-ventilated group of men. Additionally, a significant negative correlation exists between disease risk and ventilation conditions among adolescents across different age groups (*p* < 0.05).

**Table 5:** Subgroup interaction sheet between gender and environmental factors.

Characteristic	Overall disease		Acute disease		Chronic disease		Other disease	
	OR(95%CI)	*P* for interaction	OR(95%CI)	*P* for interaction	OR(95%CI)	*P* for interaction	OR(95%CI)	*P* for interaction
Ventilation		0.043		0.129		0.028		0.678
Sex.1_ventilation.1	Ref		Ref		Ref		Ref	
Sex.2_ventilation.1	0.99(0.63,1.55)		1.12(0.69,1.83)		1.26(0.56,2.81)		0.77(0.21,2.79)	
Sex.1_ventilation.2	0.71(0.50,1.01)		0.73(0.48,1.10)		1.37(0.71,2.63)		0.56(0.20,1.56)	
Sex.2_ventilation.2	0.46(0.32,0.67)***		0.59(0.39,0.89)*		0.41(0.19,0.87)*		0.18(0.05,0.69)*	
Sex.1_ventilation.3	0.58(0.40,0.85)**		0.57(0.37,0.90)*		1.03(0.50,2.11)		0.74(0.25,2.18)	
Sex.2_ventilation.3	0.62(0.43,0.91)***		0.74(0.48,1.16)		0.62(0.29,1.34)		0.32(0.09,1.15)	
Sex.1_ventilation.4	0.53(0.37,0.76)***		0.50(0.33,0.76)**		0.93(0.47,1.84)		0.55(0.19,1.57)	
Sex.2_ventilation.4	0.48(0.34,0.68)***		0.62(0.41,0.93)*		0.56(0.28,1.13)		0.38(0.13,1.12)	
Oil_fumes		0.037		0.044		0.040		0.022
Sex.1_oil_fumes.1	Ref		Ref		Ref		Ref	
Sex.2_oil_fumes.1	1.23(0.91,1.65)		1.47(1.07,2.02)*		1.08(0.61,1.90)		0.56(0.18,1.68)	
Sex.1_oil_fumes.2	2.29(1.77,2.95)***		1.91(1.44,2.55)***		2.29(1.43,3.67)***		1.99(0.90,4.38)	
Sex.2_oil_fumes.2	2.69(1.31,3.18)***		1.76(1.33,2.33)***		1.04(0.62,1.73)		0.80(0.33,1.97)	
Sex.1_oil_fumes.3	5.39(3.53,8.23)***		2.48(1.54,3.99)***		4.48(2.33,8.60)***		5.06(1.73,14.79)**	
Sex.2_oil_fumes.3	6.57(2.86,9.31)***		4.05(2.46,6.66)***		1.36(0.55,3.39)		3.91(1.11,13.81)	
Sex.1_oil_fumes.4	8.19(3.29,20.37)***		2.95(1.08,8.02)*		4.19(1.14,15.36)*		5.60(1.06,29.65)*	
Sex.2_oil_fumes.4	9.25(2.82,30.39)***		4.33(1.15,16.29)*		1.80(0.28,11.68)		17.77(3.22,98.00)***	
DM		0.904		0.551		0.361		0.685
Sex.1_dm.0	Ref		Ref		Ref		Ref	
Sex.2_dm.0	0.83(0.70,0.97)*		1.11(0.93,1.32)		0.48(0.35,0.66)***		0.51(0.30,0.88)*	
Sex.1_dm.1	2.90(2.07,4.05)***		1.75(1.21,2.55)**		1.31(0.77,2.22)		0.37(0.11,1.30)	
Sex.2_dm.1	2.33(1.72,3.15)***		1.68(1.20,2.36)**		1.11(0.67,1.84)		0.30(0.08,1.10)	

Sex 1: male, sex 2: female; ventilation 1, 2, 3, 4: no ventilated, 1 ~ 2 days/week, 3 ~ 4 days/week, >5 days/week; oil fumes 1, 2, 3, 4: no oil fume, light oil fumes, moderate oil fumes, heavy oil fumes; dm 0, 1: no, yes. ***P < 0.0001, **P < 0.001, *P < 0.05.

**Table 6:** Subgroup interaction sheet between age group and environmental factors.

Characteristic	Overall disease		Acute disease		Chronic disease		Other disease	
	OR(95%CI)	*P* for interaction	OR(95%CI)	*P* for interaction	OR(95%CI)	*P* for interaction	OR(95%CI)	*P* for interaction
Ventilation		0.049		0.012		0.025		0.565
Age_group.1_ventilation.1	Ref		Ref		Ref		Ref	
Age_group.2_ventilation.1	0.95(0.48,1.88)		0.97(0.51,1.87)		1.28(0.28,5.91)		6.34(0.65,62.32)	
Age_group.3_ventilation.1	1.61(0.81,3.18)		1.50(0.80,2.81)		5.71(1.53,21.33)**		2.49(0.24,25.73)	
Age_group.1_ventilation.2	0.56(0.33,0.97)*		0.63(0.35,1.16)		0.34(0.06,1.86)		NA	
Age_group.2_ventilation.2	0.55(0.30,1.03)		0.58(0.33,1.03)		1.21(0.32,4.61)		2.04(0.20,20.32)	
Age_group.3_ventilation.2	1.07(0.56,2.03)		1.01(0.57,1.81)		5.87(1.61,21.33)**		1.98(0.19,20.35)	
Age_group.1_ventilation.3	0.65(0.37,1.13)		0.74(0.59,0.99)*		0.40(0.27,0.68)***		0.45(0.02,8.63)	
Age_group.2_ventilation.3	0.58(0.31,1.10)		0.62(0.34,1.13)		1.72(0.44,6.66)		2.91(0.28,29.66)	
Age_group.3_ventilation.3	1.00(0.52,1.94)		0.81(0.72,0.91)*		4.63(1.23,17.48)*		2.76(0.26,29.36)	
Age_group.1_ventilation.4	0.32(0.18,0.56)***		0.38(0.20,0.71)**		0.22(0.13,0.40)***		NA	
Age_group.2_ventilation.4	0.60(0.33,1.10)		0.63(0.36,1.10)		1.59(0.43,5.85)		2.39(0.24,23.24)	
Age_group.3_ventilation.4	0.85(0.45,1.61)		0.71(0.59,0.83)**		3.46(0.93,12.82)		2.26(0.22,22.99)	
Oil_fumes		0.188		0.026		0.719		NA
Age_group.1_oil_fumes.1	Ref		Ref		Ref		Ref	
Age_group.2_oil_fumes.1	1.67(1.00,2.80)		1.71(1.14,2.56)**		3.88(1.29,11.62)*		NA	
Age_group.3_oil_fumes.1	2.61(1.51,4.51)***		2.07(1.35,3.19)***		9.77(3.39,28.23)***		NA	
Age_group.1_oil_fumes.2	2.61(1.78,3.81)***		2.27(1.53,3.37)***		1.34(0.39,4.65)		NA	
Age_group.2_oil_fumes.2	2.64(1.62,4.32)***		2.14(1.49,3.07)***		4.74(1.69,13.31)**		NA	
Age_group.3_oil_fumes.2	4.11(2.45,6.91)***		2.82(1.93,4.11)***		16.00(5.79,44.22)***		NA	
Age_group.1_oil_fumes.3	4.82(2.15,10.81)***		4.05(1.74,9.42)**		NA		NA	
Age_group.2_oil_fumes.3	5.45(2.87,10.32)***		3.17(1.77,5.69)***		8.94(2.62,30.56)***		NA	
Age_group.3_oil_fumes.3	13.80(7.25,26.28)***		5.72(3.29,9.93)***		19.89(6.42,61.63)***		NA	
Age_group.1_oil_fumes.4	9.11(1.14,32.80)		0.69(0.04,11.45)		NA		NA	
Age_group.2_oil_fumes.4	8.94(3.05,26.23)***		2.91(0.92,9.27)*		7.39(1.03,53.22)*		NA	
Age_group.3_oil_fumes.4	13.74(3.31,35.99)***		11.42(2.97,43.90)***		34.85(6.28,193.31)***		NA	
DM		0.002		0.02		0.631		0.398
Age_group.1_dm.0	Ref		Ref		Ref		Ref	
Age_group.2_dm.0	1.34(0.89,2.01)		1.18(0.94,1.49)		3.84(1.90,7.79)***		23.25(3.11,173.92)**	
Age_group.3_dm.0	2.04(1.32,3.15)**		1.45(1.13,1.86)**		11.10(5.56,22.16)***		18.89(2.48,143.84)**	
Age_group.1_dm.1	3.05(1.92,4.86)***		1.99(1.20,3.31)**		1.24(0.25,6.14)		3.92(0.19,79.58)	
Age_group.2_dm.1	2.66(1.61,4.38)***		1.50(1.01,2.25)*		6.05(2.56,14.28)***		8.82(0.85,91.77)	
Age_group.3_dm.1	10.65(5.90,19.21)***		4.13(2.54,6.70)***		23.92(10.38,55.13)***		8.34(0.72,96.68)	

Age group 1, 2, 3: “0 ~ 14,” “15 ~ 64,” “65~”; ventilation 1, 2, 3, 4: no ventilated, 1 ~ 2 days/week, 3 ~ 4 days/week, >5 days/week; oil fumes 1, 2, 3, 4: no oil fume, light oil fumes, moderate oil fumes, heavy oil fumes; DM 0, 1: no, yes. ***P < 0.0001, **P < 0.001, *P < 0.05.

Interactions exist among age groups, gender, season, respirable particulate matter, and toxic gasses in outdoor environments. Women and individuals in young to middle-aged demographics are particularly susceptible to the detrimental effects of inhalable particulate matter and toxic gasses concerning acute respiratory diseases. Notably, individuals age 15 to 64 experience a reduced risk of respiratory disease during warmer seasons. In the context of chronic respiratory diseases, women and individuals older than 14 years are more vulnerable to the hazards posed by particulate matter and toxic gasses.

## 4 Discussion

To our knowledge, this study is the first to investigate the relationship between personal habits, environmental factors, and various determinants of respiratory disease among permanent residents in southwest China. This study found that smoking, passive smoking, alcohol abuse, and other harmful lifestyle habits continue to be the primary risk factors for respiratory diseases (encompassing both acute and chronic conditions). The data for this study is updated in a timely manner, extensive in scale, and encompasses a large number of participants. It is, to a certain extent, representative of respiratory disease research in southwest China. The study employs scientific and rigorous analytical methods as well as analytical techniques, utilizing interactions for data mining, which contributes to its validity, novelty, and practicality.

The resident population in our study primarily consists of individuals from urban areas and suburbs in southwestern China. In epidemiological studies, the presence of selection bias is a concern. To enhance the validity and generalizability of our research, we strive to include a diverse representation of all age groups and genders during data collection. Another challenge in cross-sectional studies is that data is often collected through questionnaires without accompanying clinical measurements, which can easily result in recall bias and information bias. In our research, we aim to reduce the time frame for problem setting to within 5 years. Most problems exist objectively or have been defined to minimize subjective impact. All questionnaires were collected through face-to-face interactions, and the interviewers received professional training to minimize recall bias. Socioeconomic status (SES) is associated with housing conditions and the respiratory health of residents. Unfortunately, we did not collect relevant data on household housing area and lot size to accurately reflect housing conditions. Nevertheless, we adjusted for four proxy variables related to socioeconomic status—namely, personal income, education level, occupation, and type of medical insurance (19)—along with other significant potential confounding factors in all calculations. In conclusion, our findings appear to be minimally influenced by recall bias, information bias, and socioeconomic status. However, the cross-sectional design of this study restricts our ability to draw causal inferences, and the possibility of reverse causation cannot be ruled out. Additionally, a limitation of this study is that the analysis was conducted solely at the individual level.

This study employed lasso-logistic regression to identify personal behaviors that may serve as risk factors for respiratory diseases. Active smoking, exposure to secondhand smoke, alcohol consumption, a preference for salty foods, and frequent use of velvet products were all significantly associated with respiratory diseases (both acute and chronic respiratory disease). Indoor exposure to significant amounts of oil smoke, dust, and dust mites, as well as inhalable particles and toxic gasses, may pose a risk factor for respiratory diseases. The presence of sufficient natural light indoors, the availability of air conditioning during the summer months, the use of natural gas as a cooking fuel, regular monthly cleaning and disinfection of the home, and the occurrence of warm seasons may serve as protective factors. Individuals with a history of major respiratory diseases and allergies, as well as a family history of respiratory conditions, may be at an increased risk for the disease. Furthermore, negative mood states are also considered a risk factor and exhibit a positive correlation with the disease.

Based on the multivariable regression model, this study innovatively established a multi-dimensional nomogram for screening respiratory diseases, incorporating factors such as personal habits, indoor and outdoor environmental conditions, and personal disease history. This nomogram can quickly, intuitively, and accurately predict the probability of outcomes (20, 21). In both the training and validation sets, the nomogram demonstrated strong predictive performance, indicating its clinical practicality and generalizability. The nomogram model developed in this study can assist clinicians in more accurately assessing the disease risk of individual patients and providing personalized risk assessments, which is particularly crucial for early identification and intervention. This study also distinguishes between acute and chronic respiratory diseases. Furthermore, the model serves as a valuable educational tool for patients, helping them comprehend their risks and thereby motivating them to adopt healthier lifestyles to mitigate their disease risk. Additionally, the study addresses significant personal habits and environmental factors. However, potential sources of error warrant careful consideration. The model may not fully account for certain important confounding variables, such as genetic factors and socioeconomic status, which could impact its external validity and predictive accuracy. Moreover, applying the same nomogram model across different regions or populations may yield varying results, reflecting the diverse manifestations of disease and risk factors influenced by distinct environmental and cultural contexts. Despite these limitations, nomogram models retain significant clinical value in integrating complex risk factors and advancing personalized medicine. Future research should focus on incorporating additional candidate regions and predictors by increasing the sample size and broadening the scope of the study to enhance the overall applicability of the nomogram.

This study demonstrates that current smokers pose a greater risk to the respiratory system compared to non-smokers and former smokers. Additionally, we observed stronger associations between smoking-related diseases and specific demographic groups through interactions, particularly among women, adolescents, and older adults. Similar to the study by Amaral et al., the correlation between airflow obstruction and smoking status is more pronounced in women, with notable gender differences observed among current smokers exposed to the same dosage (22). This may be attributed to the generally smaller lung airways in women, which can result in increased respiratory resistance when inhaling smoke, potentially leading to difficulties in breathing. Research indicates that C-reactive protein plays a distinct role in mediating the relationship between smoking and lung function across genders. This variation may be attributed to differences in the extent to which C-reactive protein influences and individuals’ susceptibility to inflammatory processes in males and females (23). Ólafsdóttir’s study also confirms the existence of gender differences in the association between systemic inflammation and reduced lung function (24). Previous reviews have indicated that variations in specific hormone levels may influence women’s reliance on tobacco and their challenges in cessation (25). Smoking disrupts the normal growth and development of the respiratory system in adolescents. Research has demonstrated that early smoking can result in diminished lung function and heighten the risk of future respiratory diseases among this age group (26). This study demonstrates that alcohol consumption significantly influences respiratory diseases, aligning with the findings of Ng et al. (27). Our multivariate and interaction analyses revealed a positive association between the frequency of alcohol consumption and the risk of respiratory disease in women. Notably, women exhibit a higher risk than men at equivalent levels of alcohol exposure. A national epidemiological survey in the United States reveals an increase in drinking rates among women, and it indicates that the association between alcohol consumption and respiratory disease is more pronounced in women than in men (28). Experiments have demonstrated that long-term alcohol consumption can activate the oxidative stress pathway in alveolar macrophages (AMs), thereby impairing their immune function and pathogen clearance capabilities (29). The issue of second-hand smoke has garnered significant attention from society. The results of this study indicate that both adolescents and older adults are adversely affected by secondhand smoke, particularly from smoking within the household. While smoking areas and bans have been implemented in numerous public places in China, achieving some success (30), smoking bans in private residences remain ineffective. Consequently, there is a pressing need for enhanced health education targeting indoor smokers, particularly in households with older adult individuals and children. Additionally, the implementation of more effective strategies to prevent indoor smoking is essential (31). Our findings suggest that regular exercise and a lighter diet may serve as protective factors against respiratory diseases. A lighter diet can help to eliminate high-salt, high-sugar, and heavy-fat foods, as well as potential allergens that may irritate the respiratory tract. The frequency of exercise per week is inversely correlated with the incidence of respiratory diseases. This relationship may be attributed to the fact that regular physical activity enhances the body’s resistance and fortifies the heart and lungs. Furthermore, exercise can mitigate the adverse effects of stress, anxiety, and depression on the respiratory system—such as hyperventilation and inadequate lung tension—thereby indirectly promoting the homeostasis of the respiratory system. In this study, the results of the interaction analysis indicated that exercise had a more significant effect on the respiratory health of women. This may be attributed to women’s preference for gentle and sustained forms of exercise, such as yoga and brisk walking. Such activities provide pronounced benefits to the respiratory system, as they emphasize the rhythm and depth of breathing. Social and psychological pressures faced by adolescents and women may contribute to their earlier exposure to tobacco and alcohol. On one hand, social norms and peer influence can lead adolescents to perceive smoking and drinking as acceptable social behaviors (32). On the other hand, women often experience heightened emotional and social pressures, which may make them more likely to use smoking and drinking as coping mechanisms for anxiety or depression (33). Consequently, adjusting our mindset, regulating our emotions, alleviating psychological stress, and fostering an optimistic and positive attitude are highly beneficial (34) for our physical health and can significantly enhance our quality of life.

This study found that indoor natural ventilation, occurring at least once a week, is beneficial for the respiratory health of various subgroups, particularly among women and adolescents. Natural ventilation in the home can mitigate the effects of indoor dust, smoke, dust mites, and other substances on the respiratory system. This area is situated in southern China, characterized by relatively high temperatures and humidity levels. Such a warm and humid indoor environment can facilitate the growth of bacteria, mold, and other microorganisms. Previous reviews have indicated that mold odors in the home may serve as a risk factor for both rhinitis (35) and asthma (36). Regular home ventilation can effectively reduce indoor particulate matter, kitchen fume levels, and humidity. Our study found that kitchen fumes are relatively more harmful to women, which aligns with the findings of a cohort study conducted in Taiwan. Furthermore, there is a dose–response relationship between exposure to fumes from traditional Chinese cooking and the incidence of lung cancer (37). In the context of the indoor environment, it is essential to prioritize self-cleaning and home disinfection practices, enhance indoor ventilation, utilize clean energy sources for cooking, minimize exposure to environmental tobacco smoke (ETS), prevent moisture and mildew accumulation, and regularly clean indoor items (12). Regular exposure of bedding to sunlight is an effective method for reducing bioaerosols in moist mattresses, particularly in decreasing exposure to dust mites that are frequently found in bedding dust samples from Chinese households (13).

In the outdoor environment, 17.11% of respondents reported the presence of garbage stations, major traffic streets, construction sites, and other locations within 100 m of their residence. These findings suggest that such locations may influence the incidence of respiratory diseases within the population and may have interactive effects. Furthermore, the respiratory health of men, teenagers, and the older adult appears to be more significantly impacted by their surrounding environment. Several studies conducted in developed countries have identified associations between increased urbanization and the prevalence of upper respiratory tract diseases, including asthma and rhinitis, in adults (38, 39). Additionally, prior research in China has established a correlation between residing near heavily trafficked arteries and the incidence of rhinitis (40). Such environments are likely to produce ammonia (NH_3_), volatile organic compounds (VOCs), and other irritating substances. Prolonged exposure to these pollutants may result in diminished lung function and an increased risk of respiratory illnesses. The air tends to be dry during the cold season, which heightens the likelihood of acute respiratory diseases, such as influenza and pneumonia, thereby exacerbating the challenges of disease prevention and control. Therefore, it is essential to wear medical or surgical protective masks properly during the cold season and to receive timely influenza vaccinations to mitigate the spread of respiratory viruses (41).

Numerous studies have identified genes associated with the incidence of asthma, chronic obstructive pulmonary disease (COPD), and pulmonary fibrosis (42–44). Our findings support this conclusion, indicating that individuals with a family history of respiratory disease have a higher probability of developing the condition. In particular, the older adult, whose physical functions may have deteriorated, require regular physical examinations to enhance their quality of life. To enhance the external validity of the study, we plan to establish a multi-period follow-up framework in future research to analyze the dynamic relationship between various factors and respiratory diseases. This will involve regularly tracking participants’ living habits and environmental exposures. Additionally, we will refine our data collection methods by employing a diverse array of tools, including both online and offline questionnaires, long-term health monitoring, and environmental assessments, to ensure the comprehensiveness and accuracy of the data. The implementation of this design will facilitate a deeper understanding of the interactions among different factors and their long-term effects on respiratory health, thereby providing a more robust scientific foundation for the development of public health policies.

In summary, the prevalence of respiratory diseases among permanent residents in southwest China is notably high. Smoking, passive smoking, alcoholism, and other detrimental lifestyle habits remain the primary risk factors contributing to these diseases. Engaging in regular exercise, practicing relaxation techniques, managing emotions, and effectively alleviating mental stress contribute to the overall health of the respiratory system. Regular cleaning and disinfection of the home, management of kitchen fumes, cleaning of indoor items, frequent exposure of bedding and down products to sunlight, proper use of masks when outdoors, attention to personal hygiene, and routine physical examinations are all beneficial measures. Women, adolescents and the older adult are still the vulnerable groups to focus on.

## Data Availability

The datasets presented in this article are not readily available because the data has not been made public and is classified. Requests to access the datasets should be directed to HengYu Su, 526999839@qq.com.

## Footnotes

^1^ http://www.nhc.gov.cn/

## Glossary

RD: respiratory disease
Psmoke: passive smoke
FD: food taste
TOD: Type of diet
History of RD: history of respiratory disease
14PHB: Whether there is hospitalized for severe respiratory diseases such as pneumonia and bronchitis before the age of 14 years
SFOD: Suffer from other disease
CHD: coronary heart disease
Other_CVD: other cardiovascular disease
Expectoration: number of sputum expectorations per day without a cold
X ray: The number of X rays received since birth
Ventilation: housing ventilation
Lighting: Lighting condition of housing
Clearance: Housing clearance situation
Oil fumes: Household oil fumes in the past 5 years
Air condition: use of air conditioning in summer
Uheating: underfloor heating
Electric: use electric furnace in winter
Fluffy products: person and household use of fluffy products
Pet: families feed long haired pets
Cook: cooking experience in the past 5 years
Coal: Cooking with coal
Workplace: whether the workplace is a public place in the past 5 years
Surrounding: there is a factory, construction site or traffic main road within 100 m from the place of living
DF: dust fume
DM: dust mite
Emotion: emotional status in the past 5 years
Stress: stress in the past 5 years
Sleep: sleep quality in the past 5 years
