# Supplementary material for: Associations between lifestyle habits, environmental factors and respiratory diseases: a cross-sectional study from southwest China
Source: Front Public Health. 2025 Mar 5;13:1513926. doi: 10.3389/fpubh.2025.1513926 (PMC11919831; doi:10.3389/fpubh.2025.1513926)
Supplement: Supplementary file 1 [file Data_Sheet_1.pdf]

Associations between lifestyle habits, environmental factors and respiratory diseases: a cross-sectional study from southwest China

Hengyu Su[1] **Huifang Xie**[1]

Address：Xinjiang Medical University (Xuelianshan Campus) is located at No. 567, Shangde North Road, in the Shuimogou District of Urumqi City, within the Xinjiang Uygur Autonomous Region, China, with a postal code of 830017.

Hengyu Su

E-mail：526999839@qq.com

Huifang Xie

E-mail：xhfworld@sina.com

Table.S1 2024.2-2024.5 Basic information of personal habits and diseases of permanent residents in a certain area....3
Table.S2 2024.2-2024.5 Basic information of indoor environmental factors and diseases of permanent residents in a certain 5
Table.S3 2024.2-2024.5 Basic information of outdoor environmental factors and diseases of permanent residents in a certain area....8
Table.S4 2024.2-2024.5 Personal history of respiratory diseases of permanent residents in a certain area....9
Table.S5 2024.2-2024.5 Personal history of family history of permanent residents in a certain area....11
Table.S6 2024.2-2024.5 Personal history of other basic factors and diseases of permanent residents in a certain area....13
Table.S7 2024.2-2024.5 Factors influencing respiratory diseases of permanent residents in a region were selected by lasso regression....14
Figure.S1 nomogram of predicted acute respiratory disease risk established after lasso-logistic regression analysis....15
Figure.S2 ROC curve and AUC of the area under the curve of the training set and validation set in the nomogram for predicting acute respiratory diseases, calibration curve H-L and clinical decision curve DCA....15
Table.S8 Results of the confusion matrix for the nomogram for predicting acute respiratory diseases....15
Figure.S3 nomogram of predicted chronic respiratory disease risk established after lasso-logistic regression analysis....16
Figure S4 ROC curve and AUC of the area under the curve of the training set and validation set in the nomogram for predicting chronic respiratory diseases, calibration curve H-L and clinical decision curve DCA....16
Table.S9 Results of the confusion matrix for the nomogram for predicting chronic respiratory diseases....16
Table.S10 Subgroup interaction sheet between gender and lifestyle factors....17
Table.S11 Subgroup interaction sheet between age group and lifestyle factors....17
Table.S12 Subgroup interaction sheet between gender and environmental factors....18
Table.S13 Subgroup interaction sheet between age group and environmental factors....20
Table.S14 Subgroup interaction sheet between gender and disease history factors....24
Table.S15 Subgroup interaction sheet between age group and disease history factors....26
Table.S16 Subgroup interaction sheet between gender and family history factors....28
Table.S17 Subgroup interaction sheet between age group and family history factors....29
Table.S18 Subgroup interaction sheet between gender and other factors....30
Table.S19 Subgroup interaction sheet between age group and other factors....31

Table S1 2024.2-2024.5 Basic information of personal habits and diseases of permanent residents in a certain area

Variables	Not sick (n = 3551)	Sicken (n = 956)	Sicken			Total (n = 4507)
			Acute(n=782)	Chronic(n=238)	Other(n=71)	
Mask, n(%)						
No wear	2120 (59.70)	635 (66.42)	506 (64.71)	175 (73.53)	44 (61.97)	2755 (61.13)
1~2 Days / week	825 (23.23)	196 (20.50)	169 (21.61)	36 (15.13)	15 (21.13)	1021 (22.65)
3~4 Days / week	316 (8.90)	72 (7.53)	59 (7.54)	16 (6.72)	11 (15.49)	388 (8.61)
> 5 Days / week	290 (8.17)	53 (5.54)	48 (6.14)	11 (4.62)	1 (1.41)	343 (7.61)
P-value	<0.0001		0.005	<0.0001	0.053	
Smoke, n(%)						
Never smoke	2945 (82.93)	324 (33.89)	295 (37.72)	48 (20.17)	12 (16.90)	3269 (72.53)
Former smoker	344 (9.69)	321 (33.58)	253 (32.35)	88 (36.97)	26 (36.62)	665 (14.75)
Now smoker	262 (7.38)	311 (32.53)	234 (29.92)	102 (42.86)	33 (46.48)	573 (12.71)
P-value	<0.0001		<0.0001	<0.0001	<0.0001	
Smoke Number, n(%)						
0 Never smoke	2945 (82.93)	324 (33.89)	295 (37.72)	48 (20.17)	12 (16.90)	3269 (72.53)
<10 cigarettes/day	363 (10.22)	193 (20.19)	148 (18.93)	57 (23.95)	19 (26.76)	556 (12.34)
10~19 cigarettes/day	203 (5.72)	262 (27.41)	200 (25.58)	83 (34.87)	19 (26.76)	465 (10.32)
>20 cigarettes/day	40 (1.13)	177 (18.51)	139 (17.77)	50 (21.01)	21 (29.58)	217 (4.81)
P-value	<0.0001		<0.0001	<0.0001	<0.0001	
Psmoke, n(%)						
No exposed	2144 (60.38)	221 (23.12)	190 (24.30)	45 (18.91)	17 (23.94)	2365 (52.47)
1~2 Days / week	1074 (30.25)	410 (42.89)	341 (43.61)	102 (42.86)	29 (40.85)	1484 (32.93)
3~4 Days / week	266 (7.49)	223 (23.33)	174 (22.25)	59 (24.79)	15 (21.13)	489 (10.85)
Daily exposure	67 (1.89)	102 (10.67)	77 (9.85)	32 (13.45)	10 (14.08)	169 (3.75)
P-value	<0.0001		<0.0001	<0.0001	<0.0001	
Drink, n(%)						
No drinking	2795 (78.71)	518 (54.18)	508 (64.96)	127 (53.36)	34 (47.89)	3313 (73.51)
Light drinking(1 time/week)	632 (17.80)	233 (24.37)	206 (26.34)	79 (33.19)	24 (33.80)	865 (19.19)
Moderate drinking(2~3times/week)	110 (3.10)	123 (12.87)	58 (7.42)	22 (9.24)	7 (9.86)	233 (5.17)
Heavy drinking(4~times/week)	14 (0.39)	82 (8.58)	10 (1.28)	10 (4.20)	6 (8.45)	96 (2.13)
P-value	<0.0001		<0.0001	<0.0001	0.701	
Sport, n(%)						
Never exercise	1186 (33.40)	412 (43.10)	320 (40.92)	117 (49.16)	28 (39.44)	1598 (35.46)

Variables	Not sick (n = 3551)	Sicken (n = 956)	Sicken			Total (n = 4507)
			Acute(n=782)	Chronic(n=238)	Other(n=71)	
1~2 Days / week	1407 (39.62)	375 (39.23)	322 (41.18)	84 (35.29)	25 (35.21)	1782 (39.54)
3~4 Days / week	523 (14.73)	116 (12.13)	98 (12.53)	22 (9.24)	12 (16.90)	639 (14.18)
> 5 Days / week	435 (12.25)	53 (5.54)	42 (5.37)	15 (6.30)	6 (8.45)	488 (10.83)
P-value	<0.0001		<0.0001	<0.0001	0.011	
Fd, n(%)						
taste is light	809 (22.78)	229 (23.95)	191 (24.42)	58 (24.37)	19 (26.76)	1038 (23.03)
taste is balance	2464 (69.39)	596 (62.34)	494 (63.17)	133 (55.88)	39 (54.93)	3060 (67.89)
tastet is salty	278 (7.83)	131 (13.70)	97 (12.40)	47 (19.75)	13 (18.31)	409 (9.07)
P-value	<0.0001		<0.0001	<0.0001	<0.0001	
Tod, n(%)						
mainly vegetarian	503 (14.17)	163 (17.05)	132 (16.88)	45 (18.91)	14 (19.72)	666 (14.78)
balanced diet	2614 (73.61)	607 (63.49)	503 (64.32)	141 (59.24)	40 (56.34)	3221 (71.47)
mainly meat	434 (12.22)	186 (19.46)	147 (18.80)	52 (21.85)	17 (23.94)	620 (13.76)
P-value	<0.0001		<0.0001	<0.0001	0.011	

Psmoke:passive smoke ; FD: food taste; Tod:type of diet

Table S2 2024.2-2024.5 Basic information of indoor environmental factors and diseases of permanent residents in a certain

Variables	Not sick (n = 3551)	Sicken (n = 956)	Sicken			Total (n = 4507)
			Acute(n=782)	Chronic(n=238)	Other(n=71)	
Ventilation, n(%)						
No ventilated	261 (7.35)	118 (12.34)	99 (12.66)	30 (12.61)	13 (18.31)	379 (8.41)
1~2 days / week	1007 (28.36)	271 (28.35)	220 (28.13)	74 (31.09)	16 (22.54)	1278 (28.36)
3~4 days / week	721 (20.30)	197 (20.61)	160 (20.46)	48 (20.17)	16 (22.54)	918 (20.37)
> 5 days / week	1562 (43.99)	370 (38.70)	303 (38.75)	86 (36.13)	26 (36.62)	1932 (42.87)
P-value	<0.0001		<0.0001	0.035	0.018	
Lighting, n(%)						
Insufficient lighting	273 (7.69)	129 (13.49)	104 (13.30)	31 (13.03)	11 (15.49)	402 (8.92)
Good lighting	3278 (92.31)	827 (86.51)	678 (86.70)	207 (86.97)	60 (84.51)	4105 (91.08)
P-value	<0.0001		<0.0001	0.022	0.05	
Clearance, n(%)						
No clearance	1969 (55.45)	667 (69.77)	532 (68.03)	177 (74.37)	44 (61.97)	2636 (58.49)
1~2 times / month	1195 (33.65)	224 (23.43)	192 (24.55)	44 (18.49)	20 (28.17)	1419 (31.48)
3~4 times / month	258 (7.27)	45 (4.71)	40 (5.12)	11 (4.62)	5 (7.04)	303 (6.72)
> 5 times / month	129 (3.63)	20 (2.09)	18 (2.30)	6 (2.52)	2 (2.82)	149 (3.31)
P-value	<0.0001		<0.0001	<0.0001	0.956	
Oil Fumes, n(%)						
No oil fume	1314 (37.00)	220 (23.01)	184 (23.53)	51 (21.43)	14 (19.72)	1534 (34.04)
Light oil smoke(does not affect the living environment)	2102 (59.19)	620 (64.85)	508 (64.96)	151 (63.45)	40 (56.34)	2722 (60.39)
Moderate oil smoke(causing discomfort)	121 (3.41)	97 (10.15)	75 (9.59)	29 (12.18)	11 (15.49)	218 (4.84)
Heavy oil smoke(Affect the living environment and health)	14 (0.39)	19 (1.99)	15 (1.92)	7 (2.94)	6 (8.45)	33 (0.73)
P-value	<0.0001		<0.0001	<0.0001	<0.0001	
Fluffy Product, n(%)						
No use fluffy Product	1662 (46.80)	324 (33.89)	257 (32.86)	89 (37.39)	30 (42.25)	1986 (44.06)
Small use fluffy Product(or 1 Day / week)	1211 (34.10)	383 (40.06)	320 (40.92)	92 (38.66)	23 (32.39)	1594 (35.37)
Moderate use fluffy Product(or 2~3 Days / week)	555 (15.63)	187 (19.56)	151 (19.31)	48 (20.17)	12 (16.90)	742 (16.46)
Large use fluffy Product(or > 4 Days / week)	123 (3.46)	62 (6.49)	54 (6.91)	9 (3.78)	6 (8.45)	185 (4.10)
P-value	<0.0001		<0.0001	0.003	0.106	
Cook, n(%)						
Never cooking	1316 (37.06)	266 (27.82)	225 (28.77)	54 (22.69)	17 (23.94)	1582 (35.10)
1~2 days / week	1017 (28.64)	302 (31.59)	249 (31.84)	76 (31.93)	28 (39.44)	1319 (29.27)

Variables	Not sick (n = 3551)	Sicken (n = 956)	Sicken			Total (n = 4507)
			Acute(n=782)	Chronic(n=238)	Other(n=71)	
3~4 days / week	470 (13.24)	151 (15.79)	125 (15.98)	44 (18.49)	10 (14.08)	621 (13.78)
> 5 days / week	748 (21.06)	237 (24.79)	183 (23.40)	64 (26.89)	16 (22.54)	985 (21.85)
P-value	<0.0001		<0.0001	<0.0001	0.163	
Dm, n(%)						
No	3317 (93.41)	799 (83.58)	652 (83.38)	189 (79.41)	64 (90.14)	4116 (91.32)
Yes	234 (6.59)	157 (16.42)	130 (16.62)	49 (20.59)	7 (9.86)	391 (8.68)
P-value	<0.0001		<0.0001	<0.0001	0.721	
Air Condition, n(%)						
No use air-condition	600 (16.90)	200 (20.92)	149 (19.05)	69 (28.99)	22 (30.99)	800 (17.75)
1~2 days / week	863 (24.30)	215 (22.49)	169 (21.61)	59 (24.79)	26 (36.62)	1078 (23.92)
3~4 days / week	926 (26.08)	246 (25.73)	209 (26.73)	54 (22.69)	13 (18.31)	1172 (26.00)
> 5 days / week	1162 (32.72)	295 (30.86)	255 (32.61)	56 (23.53)	10 (14.08)	1457 (32.33)
P-value	0.033		0.358	<0.0001	0.002	
Heating, n(%)						
No	3331 (93.80)	870 (91.00)	703 (89.90)	219 (92.02)	64 (90.14)	4201 (93.21)
Yes	220 (6.20)	86 (9.00)	79 (10.10)	19 (7.98)	7 (9.86)	306 (6.79)
P-value	0.002		<0.0001	0.452	0.425	
Uheating, n(%)						
No	3477 (97.92)	932 (97.49)	759 (97.06)	231 (97.06)	67 (94.37)	4409 (97.83)
Yes	74 (2.08)	24 (2.51)	23 (2.94)	7 (2.94)	4 (5.63)	98 (2.17)
P-value	0.422		0.106	0.405	0.109	
Charcoal Fire, n(%)						
No	3464 (97.55)	915 (95.71)	754 (96.42)	226 (94.96)	63 (88.73)	4379 (97.16)
Yes	87 (2.45)	41 (4.29)	28 (3.58)	12 (5.04)	8 (11.27)	128 (2.84)
P-value	0.002		0.170	0.036	<0.0001	
Electric, n(%)						
No	2862 (80.60)	762 (79.71)	630 (80.56)	189 (79.41)	49 (69.01)	3624 (80.41)
Yes	689 (19.40)	194 (20.29)	152 (19.44)	49 (20.59)	22 (30.99)	883 (19.59)
P-value	0.538		0.905	0.691	0.015	
Pet, n(%)						
No	1146 (32.27)	335 (35.04)	267 (34.14)	90 (37.82)	18 (25.35)	1481 (32.86)
Yes	2405 (67.73)	621 (64.96)	515 (65.86)	148 (62.18)	53 (74.65)	3026 (67.14)
P-value	<0.0001		0.401	0.094	0.175	

coal , n(%)							
	No	3427 (96.51)	933 (97.59)	759 (97.06)	236 (99.16)	69 (97.18)	4360 (96.74)
	Yes	124 (3.49)	23 (2.41)	23 (2.94)	2 (0.84)	2 (2.82)	147 (3.26)
	P-value	0.093		0.579	0.031	1.000	
Natural Gas, n(%)							
	No	652 (18.36)	212 (22.18)	166 (21.23)	65 (27.31)	21 (29.58)	864 (19.17)
	Yes	2899 (81.64)	744 (77.82)	616 (78.77)	173 (72.69)	50 (70.42)	3643 (80.83)
	P-value	0.008		0.108	0.001	0.025	
Electricity, n(%)							
	No	2409 (67.84)	650 (67.99)	533 (68.16)	151 (63.45)	42 (59.15)	3059 (67.87)
	Yes	1142 (32.16)	306 (32.01)	249 (31.84)	87 (36.55)	29 (40.85)	1448 (32.13)
	P-value	0.929		0.850	0.133	0.133	

Ventilation: natural ventilation or mechanical ventilation; Oil Fumes:cooking oil fumes; air condition: use air condition in summer; Uheationg: under heating; Charcoal Fire,Electric: winter heating facilities; cook: personal cooking in five years; coal,natural gas and electricity:type of fuel for cooking; DM: dust mite.

Table S3 2024.2-2024.5 Basic information of outdoor environmental factors and diseases of permanent residents in a certain area

Variables	Not sick (n = 3551)	Sicken (n = 956)	Sicken			Total (n = 4507)
			Acute(n=782)	Chronic(n=238)	Other(n=71)	
Season, n(%)						
cold season	1685 (47.45)	551 (57.64)	449 (57.42)	134 (56.30)	48 (67.61)	2236 (49.61)
warm season	1866 (52.55)	405 (42.36)	333 (42.58)	104 (43.70)	23 (32.39)	2271 (50.39)
P-value	<0.0001		<0.0001	0.034	0.002	
Surrounding, n(%)						
No	3064 (86.29)	672 (70.29)	558 (71.36)	155 (65.13)	50 (70.42)	3736 (82.89)
Yes	487 (13.71)	284 (29.71)	224 (28.64)	83 (34.87)	21 (29.58)	771 (17.11)
P-value	<0.0001		<0.0001	<0.0001	0.005	
Inhalabie particles and toxic gases, n(%)						
No	3523 (99.21)	926 (96.86)	754 (96.42)	227 (95.38)	65 (91.55)	4449 (98.71)
Yes	28 (0.79)	30 (3.14)	28 (3.58)	11 (4.62)	6 (8.45)	58 (1.29)
P-value	<0.0001		<0.0001	<0.0001	<0.0001	
Workplace, n(%)						
No	1909 (53.76)	438 (45.82)	352 (45.01)	116 (48.74)	39 (54.93)	2347 (52.07)
Yes	1642 (46.24)	518 (54.18)	430 (54.99)	122 (51.26)	32 (45.07)	2160 (47.93)
P-value	<0.0001		<0.0001	0.290	0.627	

Workplace: whether the workplace is a publice place in the last five years. Surrounding:Whether there is a garbage station, factory, traffic main road, etc. within 100m around the residence. Inhalabie particles and toxic gases: history of exposure to occupational toxic metals,dust particulate matte and dust fumes and their compounds.

Table S4 2024.2-2024.5 Personal history of respiratory diseases of permanent residents in a certain area

Variables		Not sick (n = 3551)	Sicken (n = 956)	Sicken			Total (n = 4507)
				Acute(n=782)	Chronic(n=238)	Other(n=71)	
History Of RD, n(%)							
	No	3274 (92.20)	415 (43.41)	369 (47.19)	70 (29.41)	37 (52.11)	3689 (81.85)
	Yes	277 (7.80)	541 (56.59)	413 (52.81)	168 (70.59)	34 (47.89)	818 (18.15)
	P-value	<0.0001		<0.0001	<0.0001	<0.0001	
Fourt Phb, n(%)							
	No	3298 (92.88)	597 (62.45)	481 (61.51)	140 (58.82)	47 (66.20)	3895 (86.42)
	Yes	253 (7.12)	359 (37.55)	301 (38.49)	98 (41.18)	24 (33.80)	612 (13.58)
	P-value	<0.0001		<0.0001	<0.0001	<0.0001	
Sfod, n(%)							
	No	3051 (85.92)	651 (68.10)	568 (72.63)	124 (52.10)	36 (50.70)	3702 (82.14)
	Yes	500 (14.08)	305 (31.90)	214 (27.37)	114 (47.90)	35 (49.30)	805 (17.86)
	P-value	<0.0001		<0.0001	<0.0001	<0.0001	
Hypertension, n(%)							
	No	3225 (90.82)	764 (79.92)	649 (82.99)	156 (65.55)	55 (77.46)	3989 (88.51)
	Yes	326 (9.18)	192 (20.08)	133 (17.01)	82 (34.45)	16 (22.54)	518 (11.49)
	P-value	<0.0001		<0.0001	<0.0001	0.003	
Diabetes, n(%)							
	No	3422 (96.37)	878 (91.84)	721 (92.20)	210 (88.24)	65 (91.55)	4300 (95.41)
	Yes	129 (3.63)	78 (8.16)	61 (7.80)	28 (11.76)	6 (8.45)	207 (4.59)
	P-value	<0.0001		<0.0001	<0.0001	0.201	
CHD, n(%)							
	No	3497 (98.48)	910 (95.19)	750 (95.91)	215 (90.34)	61 (85.92)	4407 (97.78)
	Yes	54 (1.52)	46 (4.81)	32 (4.09)	23 (9.66)	10 (14.08)	100 (2.22)
	P-value	<0.0001		<0.0001	<0.0001	<0.0001	
Arrhythmia, n(%)							
	No	3524 (99.24)	917 (95.92)	752 (96.16)	218 (91.60)	61 (85.92)	4441 (98.54)
	Yes	27 (0.76)	39 (4.08)	30 (3.84)	20 (8.40)	10 (14.08)	66 (1.46)
	P-value	<0.0001		<0.0001	<0.0001	<0.0001	
Arteriosclerosis, n(%)							
	No	3512 (98.90)	907 (94.87)	744 (95.14)	214 (89.92)	63 (88.73)	4419 (98.05)
	Yes	39 (1.10)	49 (5.13)	38 (4.86)	24 (10.08)	8 (11.27)	88 (1.95)
	P-value	<0.0001		<0.0001	<0.0001	<0.0001	

Variables	Not sick (n = 3551)	Sicken (n = 956)	Sicken			Total (n = 4507)
			Acute(n=782)	Chronic(n=238)	Other(n=71)	
cough , n(%)						
No expectoration	2954 (83.19)	490 (51.26)	432 (55.24)	86 (36.13)	33 (46.48)	3444 (76.41)
1-5 times / day	536 (15.09)	368 (38.49)	284 (36.32)	102 (42.86)	26 (36.62)	904 (20.06)
6-9 times / day	39 (1.10)	58 (6.07)	35 (4.48)	35 (14.71)	5 (7.04)	97 (2.15)
> 10 times / day	22 (0.62)	40 (4.18)	31 (3.96)	15 (6.30)	7 (9.86)	62 (1.38)
P-value	<0.0001		<0.0001	<0.0001	<0.0001	
Allergy history, n(%)						
No	3234 (91.07)	598 (62.55)	488 (62.40)	157 (65.97)	40 (56.34)	3832 (85.02)
Yes	317 (8.93)	358 (37.45)	294 (37.60)	81 (34.03)	31 (43.66)	675 (14.98)
P-value	<0.0001		<0.0001	<0.0001	<0.0001	

Fourt Phb:Whether there is hospitalized for severe respiratory diseases such as pneumonia and bronchitis before the age of 14 years; sfod: suffer from other disease. X_f: Family history of X. Emotion,stress,sleep:The survey period was within 5 years.

Table S5 2024.2-2024.5 Personal history of family history of permanent residents in a certain area

Variables		Not sick (n = 3551)	Sicken (n = 956)	Sicken			Total (n = 4507)
				Acute(n=782)	Chronic(n=238)	Other(n=71)	
COPD f, n(%)							
	No	3366 (94.79)	672 (70.29)	560 (71.61)	147 (61.76)	43 (60.56)	4038 (89.59)
	Yes	185 (5.21)	284 (29.71)	222 (28.39)	91 (38.24)	28 (39.44)	469 (10.41)
	P-value	<0.0001		<0.0001	<0.0001	<0.0001	
Bronchitis f, n(%)							
	No	3269 (92.06)	673 (70.40)	555 (70.97)	140 (58.82)	49 (69.01)	3942 (87.46)
	Yes	282 (7.94)	283 (29.60)	227 (29.03)	98 (41.18)	22 (30.99)	565 (12.54)
	P-value	<0.0001		<0.0001	<0.0001	<0.0001	
Asthma f, n(%)							
	No	3481 (98.03)	657 (68.72)	560 (71.61)	134 (56.30)	42 (59.15)	4138 (91.81)
	Yes	70 (1.97)	299 (31.28)	222 (28.39)	104 (43.70)	29 (40.85)	369 (8.19)
	P-value	<0.0001		<0.0001	<0.0001	<0.0001	
Lung fibrosis f, n(%)							
	No	3534 (99.52)	864 (90.38)	706 (90.28)	210 (88.24)	60 (84.51)	4398 (97.58)
	Yes	17 (0.48)	92 (9.62)	76 (9.72)	28 (11.76)	11 (15.49)	109 (2.42)
	P-value	<0.0001		<0.0001	<0.0001	<0.0001	
Pulmonary hypertension f, n(%)							
	No	3541 (99.72)	936 (97.91)	765 (97.83)	228 (95.80)	65 (91.55)	4477 (99.33)
	Yes	10 (0.28)	20 (2.09)	17 (2.17)	10 (4.20)	6 (8.45)	30 (0.67)
	P-value	<0.0001		<0.0001	<0.0001	<0.0001	
CHD f, n(%)							
	No	3413 (96.11)	891 (93.20)	735 (93.99)	211 (88.66)	57 (80.28)	4304 (95.50)
	Yes	138 (3.89)	65 (6.80)	47 (6.01)	27 (11.34)	14 (19.72)	203 (4.50)
	P-value	<0.0001		0.025	<0.0001	<0.0001	
Hypertensive f, n(%)							
	No	2990 (84.20)	744 (77.82)	610 (78.01)	170 (71.43)	50 (70.42)	3734 (82.85)
	Yes	561 (15.80)	212 (22.18)	172 (21.99)	68 (28.57)	21 (29.58)	773 (17.15)
	P-value	<0.0001		<0.0001	<0.0001	0.005	
Arrhythmia f, n(%)							
	No	3498 (98.51)	924 (96.65)	761 (97.31)	224 (94.12)	58 (81.69)	4422 (98.11)
	Yes	53 (1.49)	32 (3.35)	21 (2.69)	14 (5.88)	13 (18.31)	85 (1.89)
	P-value	<0.0001		0.071	<0.0001	<0.0001	

Variables	Not sick (n = 3551)	Sicken (n = 956)	Sicken			Total (n = 4507)
			Acute(n=782)	Chronic(n=238)	Other(n=71)	
Diabetes f, n(%)						
No	3291 (92.68)	860 (89.96)	706 (90.28)	208 (87.39)	57 (80.28)	4151 (92.10)
Yes	260 (7.32)	96 (10.04)	76 (9.72)	30 (12.61)	14 (19.72)	356 (7.90)
P-value	0.006		0.038	<0.0001	<0.0001	

Table S6 2024.2-2024.5 Personal history of other basic factors and diseases of permanent residents in a

certain area

Variables	Not sick (n = 3551)	Sicken (n = 956)	Sicken			Total (n = 4507)
			Acute(n=782)	Chronic(n=238)	Other(n=71)	
Emotion, n(%)						
Never mad	1302 (36.67)	197 (20.61)	159 (20.33)	38 (15.97)	22 (30.99)	1499 (33.26)
1~2 times / week	1940 (54.63)	585 (61.19)	476 (60.87)	156 (65.55)	37 (52.11)	2525 (56.02)
3~5 times / week	228 (6.42)	120 (12.55)	105 (13.43)	30 (12.61)	4 (5.63)	348 (7.72)
> 5 times / week	81 (2.28)	54 (5.65)	42 (5.37)	14 (5.88)	8 (11.27)	135 (3.00)
P-value	<0.0001		<0.0001	<0.0001	<0.0001	
Stress, n(%)						
No stress	1616 (45.51)	318 (33.26)	258 (32.99)	79 (33.19)	24 (33.80)	1934 (42.91)
1~2 times / month	1226 (34.53)	362 (37.87)	288 (36.83)	93 (39.08)	22 (30.99)	1588 (35.23)
3~5 times / month	398 (11.21)	133 (13.91)	121 (15.47)	26 (10.92)	11 (15.49)	531 (11.78)
> 5 times / month	311 (8.76)	143 (14.96)	115 (14.71)	40 (16.81)	14 (19.72)	454 (10.07)
P-value	<0.0001		<0.0001	<0.0001	<0.0001	
Sleep, n(%)						
Never insomnia	1786 (50.30)	310 (32.43)	270 (34.53)	58 (24.37)	18 (25.35)	2096 (46.51)
1~2 times / week	1443 (40.64)	472 (49.37)	372 (47.57)	129 (54.20)	34 (47.89)	1915 (42.49)
3~5 times / week	195 (5.49)	114 (11.92)	93 (11.89)	33 (13.87)	6 (8.45)	309 (6.86)
> 5 times / week	127 (3.58)	60 (6.28)	47 (6.01)	18 (7.56)	13 (18.31)	187 (4.15)
P-value	<0.0001		<0.0001	0.006	<0.0001	

Table S7 2024.2-2024.5 Factors influencing respiratory diseases of permanent residents in a region were selected by lasso regression

Number	Variable	Coef
1	Season	-0.298184104
2	Sex	0.408676997
3	Age_group	0.0989067988
4	Education	-0.080967878
5	Occupation	-0.066348982
6	Mask	-0.018650676
7	Smoke	0.248801692
8	Smoke_number	0.835692602
9	Psmoke	0.492089122
10	Drink	0.386535456
11	Sport	-0.131040890
12	FD	-0.210895732
13	TOD	-0.122314656
14	History_of_RD	1.5641082949
15	fourt_phb	1.762982525
16	CHD	0.553816336
17	Cough	0.403391585
18	Ventilation	-0.139243589
19	Lighting	-0.195691085
20	Oil_fumes	0.012831927
21	Air_condition	-0.070108310
22	Fluffy_product	0.009743838
23	Natural_gas	-0.016131941
24	Inhalabie particles and toxic gases	0.242109996
25	DM	0.439492020
26	Emotion	0.094001510
27	Stress	-0.027036587
28	Sleep	0.1707293725
29	Allergy_history	1.376558987
30	COPD_f	0.602359777
31	Bronchitis_f	0.631802172
32	Asthma_f	1.779691711
33	Lung_fibrosis_f	0.044861502
34	CHD_f	-0.224813015

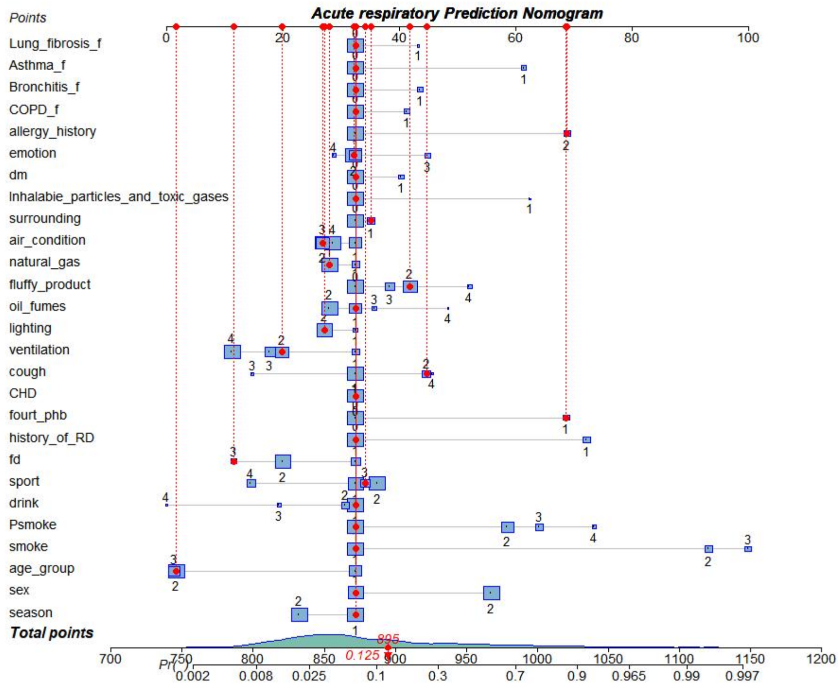

Figure S1 nomogram of predicted acute respiratory disease risk established after lasso-logistic regression analysis

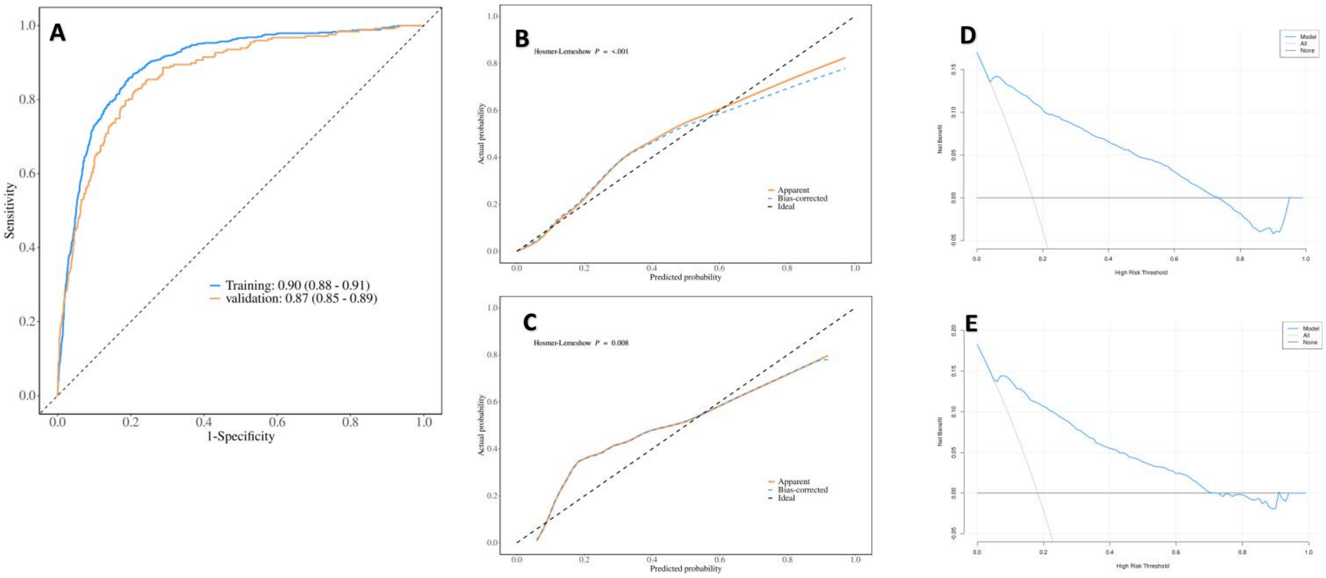

Figure S2 ROC curve and AUC of the area under the curve of the training set and validation set in the nomogram for predicting acute respiratory diseases, calibration curve H-L and clinical decision curve DCA

Table S8Results of the confusion matrix for the nomogram for predicting acute respiratory diseases

Data	AUC (95%CI)	Accuracy (95%CI)	Sensitivity (95%CI)	Specificity (95%CI)	PPV (95%CI)	NPV (95%CI)	cut off
Train	0.897 (0.882-0.911)	0.816 (0.802-0.829)	0.808 (0.793 - 0.823)	0.856 (0.826 - 0.886)	0.965 (0.957 - 0.973)	0.477 (0.445 - 0.508)	0.148
Test	0.870 (0.845-0.895)	0.803 (0.781-0.824)	0.804 (0.780 - 0.827)	0.802 (0.752 - 0.851)	0.948 (0.934 - 0.962)	0.477 (0.429 - 0.525)	0.148

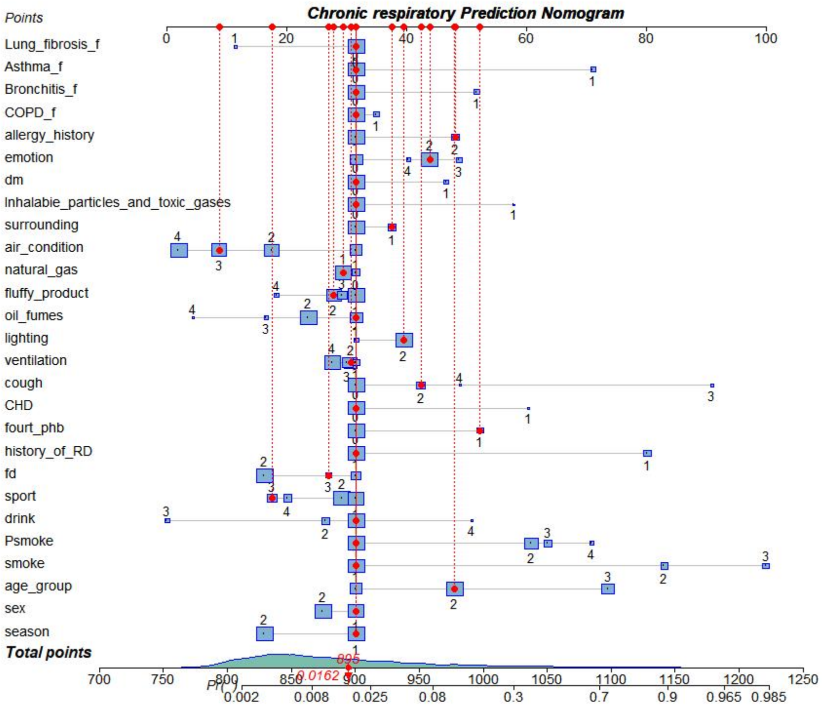

Figure S3 nomogram of predicted chronic respiratory disease risk established after lasso-logistic regression analysis

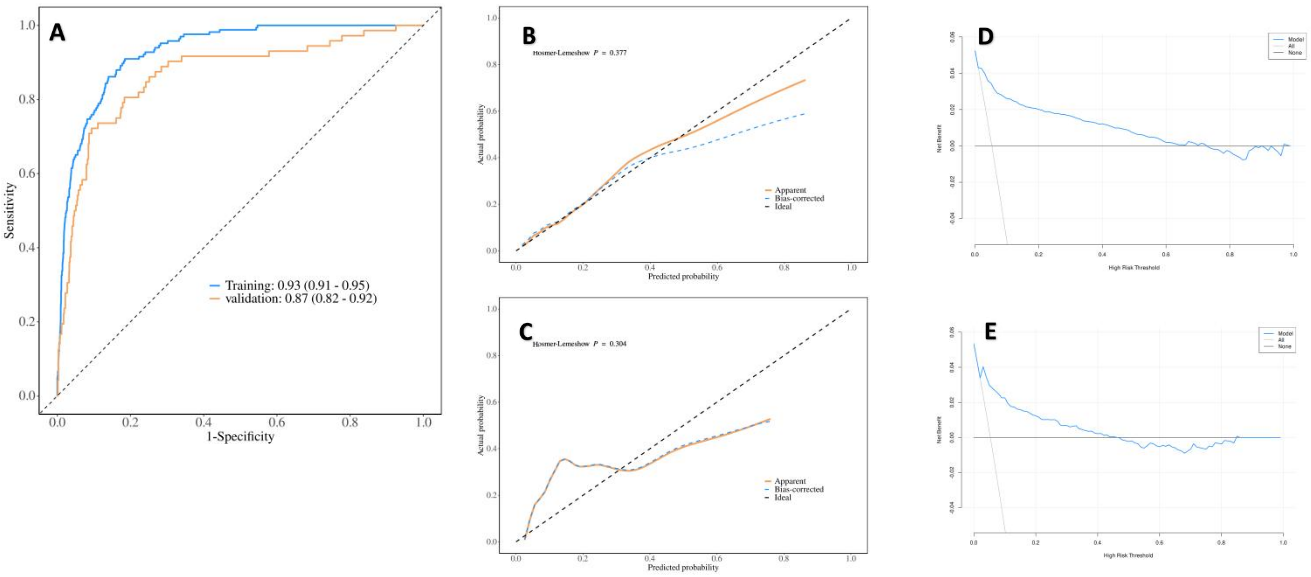

Figure S4 ROC curve and AUC of the area under the curve of the training set and validation set in the nomogram for predicting chronic respiratory diseases, calibration curve H-L and clinical decision curve DCA

Table S9 Results of the confusion matrix for the nomogram for predicting chronic respiratory diseases

Data	AUC (95%CI)	Accuracy (95%CI)	Sensitivity (95%CI)	Specificity (95%CI)	PPV (95%CI)	NPV (95%CI)	cut off
Train	0.930 (0.914-0.947)	0.819 (0.805-0.833)	0.814 (0.800 - 0.828)	0.910 (0.866 - 0.953)	0.994 (0.991 - 0.997)	0.214 (0.184 - 0.244)	0.041
Test	0.866 (0.817-0.915)	0.814 (0.793-0.835)	0.815 (0.794 - 0.836)	0.806 (0.714 - 0.897)	0.987 (0.980 - 0.994)	0.197 (0.151 - 0.242)	0.041

Table S10 Subgroup interaction sheet between gender and lifestyle factors

characteristic	Overall disease		Acute disease		Chronic disease		Other disease	
	OR(95%CI)	P for interaction	OR(95%CI)	P for interaction	OR(95%CI)	P for interaction	OR(95%CI)	P for interaction
**fd**		0.357				0.103		0.018
sex.1_fd.1	Ref		Ref		Ref		Ref	
sex.2_fd.1	1.03(0.75,1.40)		1.90(1.30,2.77)***		1.08(0.61,1.93)		1.67(0.64,4.37)	
sex.1_fd.2	0.99(0.75,1.29)		0.54(0.38,0.75)***		0.54(0.34,0.88)*		0.73(0.32,1.64)	
sex.2_fd.2	0.82(0.62,1.07)		1.36(0.97,1.91)		0.50(0.30,0.85)*		0.38(0.13,1.09)	
sex.1_fd.3	1.79(1.25,2.57)***		0.63(0.40,0.99)*		0.69(0.38,1.26)		0.60(0.20,1.78)	
sex.2_fd.3	1.29(0.85,1.97)		1.37(0.82,2.29)		1.54(0.76,3.10)		2.15(0.69,6.70)	

Table S11 Subgroup interaction sheet between age group and lifestyle factors

characteristic	Overall disease		Acute disease		Chronic disease		Other disease	
	OR(95%CI)	P for interaction	OR(95%CI)	P for interaction	OR(95%CI)	P for interaction	OR(95%CI)	P for interaction
fd		0.273		0.903				0.225
age_group.1_fd.1	Ref		Ref		Ref		Ref	
age_group.2_fd.1	1.24(0.72,2.12)		0.54(0.34,0.84)**		1.41(0.40,4.97)		3188165.83(0.00,Inf)	
age_group.3_fd.1	1.52(0.87,2.68)		0.55(0.34,0.90)**		4.14(1.21,14.13)*		1555650.56(0.00,Inf)	
age_group.1_fd.2	0.80(0.54,1.17)		0.65(0.43,0.99)*		0.73(0.19,2.82)		304045.82(0.00,Inf)	
age_group.2_fd.2	0.98(0.59,1.63)		0.29(0.19,0.44)***		0.66(0.20,2.23)		1101341.75(0.00,Inf)	
age_group.3_fd.2	1.71(1.00,2.91)*		0.37(0.24,0.58)***		2.60(0.78,8.69)		1317814.54(0.00,Inf)	
age_group.1_fd.3	1.43(0.70,2.89)		0.65(0.29,1.47)		0.00(0.00,Inf)		1983716.74(0.00,Inf)	
age_group.2_fd.3	1.48(0.83,2.65)		0.32(0.19,0.55)***		1.87(0.52,6.72)		1200071.25(0.00,Inf)	
age_group.3_fd.3	3.28(1.78,6.02)***		0.40(0.23,0.72)**		2.87(0.80,10.33)		2172324.32(0.00,Inf)	

Table S12 Subgroup interaction sheet between gender and environmental factors

characteristic	Overall disease			Acute disease			Chronic disease			Other disease		
	OR(95%CI)	P	P for interaction	OR(95%CI)	P	P for interaction	OR(95%CI)	P	P for interaction	OR(95%CI)	P	P for interaction
lighting			0.84			0.845			0.255			0.839
sex.1_lighting.1	Ref			Ref			Ref			Ref		
sex.2_lighting.1	0.82(0.53,1.27)	0.377		1.04(0.65,1.68)	0.863		0.84(0.37,1.90)	0.68		0.62(0.15,2.59)	0.51	
sex.1_lighting.2	0.54(0.39,0.73)	<0.001		0.71(0.49,1.03)	0.068		1.28(0.71,2.30)	0.414		1.32(0.50,3.48)	0.577	
sex.2_lighting.2	0.46(0.34,0.63)	<0.001		0.77(0.53,1.12)	0.179		0.65(0.35,1.19)	0.162		0.69(0.25,1.93)	0.482	
ventilation			0.043			0.129			0.028			0.678
sex.1_ventilation.1	Ref			Ref			Ref			Ref		
sex.2_ventilation.1	0.99(0.63,1.55)	0.96		1.12(0.69,1.83)	0.646		1.26(0.56,2.81)	0.577		0.77(0.21,2.79)	0.696	
sex.1_ventilation.2	0.71(0.50,1.01)	0.057		0.73(0.48,1.10)	0.132		1.37(0.71,2.63)	0.348		0.56(0.20,1.56)	0.265	
sex.2_ventilation.2	0.46(0.32,0.67)	<0.001		0.59(0.39,0.89)	0.013		0.41(0.19,0.87)	0.021		0.18(0.05,0.69)	0.012	
sex.1_ventilation.3	0.58(0.40,0.85)	0.005		0.57(0.37,0.90)	0.016		1.03(0.50,2.11)	0.941		0.74(0.25,2.18)	0.588	
sex.2_ventilation.3	0.62(0.43,0.91)	<0.001		0.74(0.48,1.16)	0.189		0.62(0.29,1.34)	0.225		0.32(0.09,1.15)	0.082	
sex.1_ventilation.4	0.53(0.37,0.76)	<0.001		0.50(0.33,0.76)	0.001		0.93(0.47,1.84)	0.836		0.55(0.19,1.57)	0.264	
sex.2_ventilation.4	0.48(0.34,0.68)	<0.001		0.62(0.41,0.93)	0.02		0.56(0.28,1.13)	0.104		0.38(0.13,1.12)	0.079	
air_condition			0.431			0.466		0.583				0.011
sex.1_air_condition.1	Ref			Ref			Ref			Ref		
sex.2_air_condition.1	0.68(0.49,0.94)	0.022		0.94(0.65,1.36)	0.74		0.42(0.24,0.73)	0.002		0.16(0.05,0.56)	0.004	
sex.1_air_condition.2	0.73(0.54,1.00)	0.048		0.69(0.48,0.99)	0.045		0.55(0.34,0.87)	0.012		0.69(0.33,1.44)	0.327	
sex.2_air_condition.2	0.62(0.46,0.85)	0.003		0.79(0.55,1.13)	0.202		0.26(0.14,0.46)	<0.001		0.43(0.18,1.02)	0.056	
sex.1_air_condition.3	0.79(0.58,1.07)	0.13		0.81(0.57,1.15)	0.243		0.37(0.22,0.61)	<0.001		0.12(0.04,0.37)	<0.001	
sex.2_air_condition.3	0.68(0.50,0.92)	0.011		0.78(0.55,1.11)	0.167		0.23(0.13,0.40)	<0.001		0.27(0.11,0.65)	0.004	
sex.1_air_condition.4	0.70(0.51,0.94)	0.019		0.71(0.50,1.01)	0.054		0.32(0.19,0.54)	<0.001		0.21(0.08,0.53)	0.001	
sex.2_air_condition.4	0.68(0.50,0.90)	0.008		0.91(0.65,1.26)	0.563		0.22(0.13,0.37)	<0.001		0.07(0.02,0.27)	<0.001	
fluffy_product			0.034			0.495		0.135			0.601	
sex.1_fluffy_product.1	Ref			Ref			Ref			Ref		
sex.2_fluffy_product.1	0.81(0.63,1.04)	0.093		1.13(0.86,1.48)	0.387		0.61(0.38,0.96)	0.033		0.65(0.30,1.43)	0.284	
sex.1_fluffy_product.2	1.47(1.16,1.86)	0.002		1.55(1.18,2.04)	0.002		1.28(0.85,1.93)	0.232		1.20(0.60,2.41)	0.609	
sex.2_fluffy_product.2	1.43(1.13,1.80)	0.003		1.87(1.44,2.44)	<0.001		0.80(0.51,1.26)	0.341		0.40(0.15,1.04)	0.061	
sex.1_fluffy_product.3	2.20(1.63,2.98)	<0.001		1.73(1.22,2.44)	0.002		1.98(1.22,3.19)	0.005		1.02(0.38,2.69)	0.972	
sex.2_fluffy_product.3	1.26(0.94,1.69)	0.116		1.55(1.13,2.14)	0.007		0.55(0.29,1.02)	0.058		0.86(0.33,2.25)	0.754	
sex.1_fluffy_product.4	3.29(1.96,5.51)	<0.001		2.95(1.69,5.15)	<0.001		0.78(0.26,2.30)	0.649		2.63(0.74,9.37)	0.135	
sex.2_fluffy_product.4	1.94(1.24,3.03)	0.004		2.41(1.49,3.90)	<0.001		0.74(0.28,1.95)	0.543		1.14(0.25,5.26)	0.862	

oil_fumes			0.037			0.044			0.04		0.29	
sex.1_oil_fumes.1	Ref			Ref			Ref			Ref		
sex.2_oil_fumes.1	1.23(0.91,1.65)	0.172		1.47(1.07,2.02)	0.018		1.08(0.61,1.90)	0.792		0.56(0.18,1.68)	0.297	
sex.1_oil_fumes.2	2.29(1.77,2.95)	<0.001		1.91(1.44,2.55)	<0.001		2.29(1.43,3.67)	<0.001		1.99(0.90,4.38)	0.088	
sex.2_oil_fumes.2	2.69(1.31,3.18)	<0.001		1.76(1.33,2.33)	<0.001		1.04(0.62,1.73)	0.886		0.80(0.33,1.97)	0.631	
sex.1_oil_fumes.3	5.39(3.53,8.23)	<0.001		2.48(1.54,3.99)	<0.001		4.48(2.33,8.60)	<0.001		5.06(1.73,14.79)	0.003	
sex.2_oil_fumes.3	6.57(2.86,9.31)	<0.001		4.05(2.46,6.66)	<0.001		1.36(0.55,3.39)	0.508		3.91(1.11,13.81)	0.034	
sex.1_oil_fumes.4	8.19(3.29,20.37)	<0.001		2.95(1.08,8.02)	0.034		4.19(1.14,15.36)	0.031		5.60(1.06,29.65)	0.043	
sex.2_oil_fumes.4	9.25(2.82,30.39)	<0.001		4.33(1.15,16.29)	0.03		1.80(0.28,11.68)	0.539		17.77(3.22,98.00)	<0.001	
dm			0.904			0.551		0.116			0.615	
sex.1_dm.0	Ref			Ref			Ref			Ref		
sex.2_dm.0	0.83(0.70,0.97)	0.022		1.11(0.93,1.32)	0.234		0.48(0.35,0.66)	<0.001		0.51(0.30,0.88)	0.016	
sex.1_dm.1	2.90(2.07,4.05)	<0.001		1.75(1.21,2.55)	0.003		1.31(0.77,2.22)	0.315		0.37(0.11,1.30)	0.121	
sex.2_dm.1	2.33(1.72,3.15)	<0.001		1.68(1.20,2.36)	0.002		1.11(0.67,1.84)	0.681		0.30(0.08,1.10)	0.069	
natural_gas			0.985			0.593		0.216				0.782
sex.1_natural_gas.0	Ref			Ref			Ref			Ref		
sex.2_natural_gas.0	0.84(0.61,1.16)	0.303		1.21(0.85,1.74)	0.292		0.39(0.22,0.70)	0.002		0.48(0.17,1.30)	0.146	
sex.1_natural_gas.1	0.83(0.65,1.06)	0.128		0.90(0.67,1.20)	0.476		0.62(0.42,0.91)	0.015		0.79(0.40,1.57)	0.499	
sex.2_natural_gas.1	0.70(0.55,0.90)	0.005		0.98(0.74,1.30)	0.881		0.36(0.24,0.55)	<0.001		0.44(0.21,0.94)	0.034	
season			0.048		0.172				0.565			0.284
sex.1_season.1	Ref			Ref			Ref			Ref		
sex.2_season.1	0.96(0.78,1.17)	0.656		1.23(0.99,1.53)	0.064		0.58(0.40,0.84)	0.004		0.65(0.35,1.19)	0.162	
sex.1_season.2	0.75(0.61,0.92)	0.006		0.71(0.56,0.91)	0.006		0.72(0.51,1.02)	0.065		0.53(0.28,0.99)	0.048	
sex.2_season.2	0.56(0.45,0.69)	<0.001		0.70(0.55,0.88)	0.002		0.35(0.23,0.53)	<0.001		0.19(0.08,0.45)	<0.001	
Inhalabie particles and toxic gases			0.617		0.017				0.009			0.777
sex.1_IPTG.0	Ref			Ref			Ref			Ref		
sex.2_IPTGl.0	0.84(0.72,0.97)	0.021		1.11(0.94,1.30)	0.231		0.52(0.39,0.69)	<0.001		0.53(0.31,0.89)	0.017	
sex.1_IPTG.1	3.29(1.50,7.22)	0.003		2.76(1.18,6.43)	0.019		1.81(1.14,2.13)	0.003		3.30(0.82,13.30)	0.092	
sex.2_IPTGl.1	3.61(1.74,7.49)	<0.001		3.91(1.81,8.45)	<0.001		2.17(1.49,3.97)	<0.001		2.36(0.44,12.59)	0.314	

IPTG:Inhalabie particles and toxic gases

Table S13 Subgroup interaction sheet between age group and environmental factors

characteristic	Overall disease			Acute disease			Chronic disease			Other disease		
	OR(95%CI)	P	P for interaction	OR(95%CI)	P	P for interaction	OR(95%CI)	P	P for interaction	OR(95%CI)	P	P for interaction
lighting			0.456			0.693			0.083			0.009
age_group.1_lighting.1	Ref			Ref			Ref			Ref		
age_group.2_lighting.1	0.84(0.44,1.60)	0.587		0.94(0.50,1.75)	0.839		0.96(0.21,4.41)	0.957		1.95(0.33,11.67)	0.463	
age_group.3_lighting.1	1.40(0.71,2.78)	0.329		1.16(0.61,2.20)	0.647		7.05(1.89,26.27)	0.004		0.67(0.09,4.98)	0.698	
age_group.1_lighting.2	0.42(0.26,0.69)	<0.001		0.61(0.35,1.07)	0.086		0.37(0.09,1.57)	0.18		0.00(0.00,Inf)	0.975	
age_group.2_lighting.2	0.51(0.29,0.92)	0.026		0.70(0.41,1.21)	0.202		1.89(0.53,6.81)	0.329		2.12(0.39,11.41)	0.381	
age_group.3_lighting.2	0.84(0.46,1.54)	0.566		0.96(0.56,1.66)	0.888		5.29(1.49,18.80)	0.01		1.98(0.37,10.56)	0.425	
ventilation			0.049			0.132			0.285			0.565
age_group.1_ventilation.1	Ref			Ref			Ref			Ref		
age_group.2_ventilation.1	0.95(0.48,1.88)	0.884		0.97(0.51,1.87)	0.936		1.28(0.28,5.91)	0.75		6.34(0.65,62.32)	0.113	
age_group.3_ventilation.1	1.61(0.81,3.18)	0.174		1.50(0.80,2.81)	0.208		5.71(1.53,21.33)	0.01		2.49(0.24,25.73)	0.443	
age_group.1_ventilation.2	0.56(0.33,0.97)	0.039		0.63(0.35,1.16)	0.137		0.34(0.06,1.86)	0.214		0.00(0.00,Inf)	0.986	
age_group.2_ventilation.2	0.55(0.30,1.03)	0.062		0.58(0.33,1.03)	0.065		1.21(0.32,4.61)	0.782		2.04(0.20,20.32)	0.543	
age_group.3_ventilation.2	1.07(0.56,2.03)	0.838		1.01(0.57,1.81)	0.963		5.87(1.61,21.33)	0.007		1.98(0.19,20.35)	0.565	
age_group.1_ventilation.3	0.65(0.37,1.13)	0.126		0.74(0.40,1.38)	0.342		0.40(0.07,2.20)	0.293		0.45(0.02,8.63)	0.593	
age_group.2_ventilation.3	0.58(0.31,1.10)	0.095		0.62(0.34,1.13)	0.116		1.72(0.44,6.66)	0.434		2.91(0.28,29.66)	0.368	
age_group.3_ventilation.3	1.00(0.52,1.94)	0.997		0.81(0.43,1.51)	0.505		4.63(1.23,17.48)	0.024		2.76(0.26,29.36)	0.399	
age_group.1_ventilation.4	0.32(0.18,0.56)	<0.001		0.38(0.20,0.71)	0.003		0.22(0.03,1.46)	0.117		0.00(0.00,Inf)	0.984	
age_group.2_ventilation.4	0.60(0.33,1.10)	0.099		0.63(0.36,1.10)	0.106		1.59(0.43,5.85)	0.486		2.39(0.24,23.24)	0.454	
age_group.3_ventilation.4	0.85(0.45,1.61)	0.618		0.71(0.39,1.28)	0.257		3.46(0.93,12.82)	0.063		2.26(0.22,22.99)	0.492	
air_condition			0.132			0.042			0.07			0.521
age_group.1_air_condition.1	Ref			Ref			Ref			Ref		
age_group.2_air_condition.1	1.53(0.84,2.82)	0.168		1.60(0.93,2.75)	0.087		3.23(0.64,16.32)	0.156		4.22(0.79,22.68)	0.093	
age_group.3_air_condition.1	2.95(1.63,5.35)	<0.001		1.83(1.11,3.02)	0.019		23.74(5.50,102.43)	<0.001		4.53(0.90,22.67)	0.066	
age_group.1_air_condi	0.88(0.51,1.51)	0.634		0.88(0.50,1.57)	0.673		0.83(0.11,6.16)	0.858		0.00(0.00,Inf)	0.988	

tion.2												
age_group.2_air_condition.2	1.31(0.73,2.36)	0.362		0.91(0.55,1.52)	0.722		4.27(0.95,19.26)	0.059		5.49(1.11,27.13)	0.037	
age_group.3_air_condition.2	2.49(1.37,4.53)	0.003		1.93(1.16,3.20)	0.011		14.11(3.21,62.00)	<0.001		3.56(0.66,19.07)	0.139	
age_group.1_air_condition.3	1.19(0.72,1.98)	0.494		1.06(0.62,1.82)	0.82		0.62(0.08,4.58)	0.638		0.00(0.00,Inf)	0.987	
age_group.2_air_condition.3	1.61(0.91,2.86)	0.101		1.37(0.84,2.22)	0.208		4.37(0.98,19.43)	0.053		1.94(0.37,10.10)	0.431	
age_group.3_air_condition.3	1.97(1.07,3.62)	0.03		1.28(0.75,2.18)	0.365		9.36(2.09,41.85)	0.003		1.12(0.17,7.56)	0.909	
age_group.1_air_condition.4	1.35(0.82,2.21)	0.235		1.40(0.83,2.37)	0.204		1.79(0.33,9.68)	0.501		0.00(0.00,Inf)	0.987	
age_group.2_air_condition.4	1.32(0.75,2.32)	0.33		1.22(0.76,1.96)	0.414		3.69(0.84,16.33)	0.085		0.96(0.18,5.29)	0.965	
age_group.3_air_condition.4	2.15(1.16,3.98)	0.016		1.61(1.03,2.76)	0.037		7.71(1.68,35.42)	0.009		1.56(0.23,10.62)	0.65	
fluffy_product			0.301			0.788			0.295			0.049
age_group.1_fluffy_product.1	Ref			Ref			Ref			Ref		
age_group.2_fluffy_product.1	1.31(0.81,2.12)	0.268		1.19(0.83,1.69)	0.337		2.72(1.15,6.44)	0.023		7.03(1.54,32.04)	0.012	
age_group.3_fluffy_product.1	2.34(1.42,3.84)	<0.001		1.67(1.17,2.40)	0.005		8.18(3.61,18.50)	<0.001		4.97(1.05,23.47)	0.043	
age_group.1_fluffy_product.2	1.85(1.26,2.71)	0.002		1.77(1.18,2.65)	0.005		0.94(0.27,3.29)	0.92		0.00(0.00,Inf)	0.987	
age_group.2_fluffy_product.2	2.20(1.37,3.52)	0.001		1.95(1.39,2.74)	<0.001		3.59(1.55,8.34)	0.003		4.16(0.86,20.05)	0.075	
age_group.3_fluffy_product.2	3.40(2.04,5.65)	<0.001		2.50(1.72,3.63)	<0.001		10.46(4.56,23.96)	<0.001		8.03(1.69,38.14)	0.009	
age_group.1_fluffy_product.3	1.68(1.03,2.76)	0.038		1.48(0.89,2.46)	0.133		0.00(0.00,Inf)	0.976		0.00(0.00,Inf)	0.991	
age_group.2_fluffy_product.3	2.21(1.34,3.65)	0.002		1.80(1.22,2.67)	0.003		3.39(1.35,8.56)	0.01		8.76(1.76,43.70)	0.008	
age_group.3_fluffy_product.3	4.85(2.79,8.44)	<0.001		2.53(1.61,3.97)	<0.001		13.16(5.48,31.58)	<0.001		3.82(0.59,24.58)	0.159	
age_group.1_fluffy_product.4	4.80(2.37,9.73)	<0.001		3.95(1.89,8.27)	<0.001		0.00(0.00,Inf)	0.988		0.00(0.00,Inf)	0.995	
age_group.2_fluffy_product.4	3.62(1.98,6.65)	<0.001		2.47(1.43,4.29)	0.001		3.70(1.10,12.43)	0.034		25.69(4.54,145.45)	<0.001	
age_group.3_fluffy_product.4	3.87(1.72,8.73)	0.001		2.89(1.35,6.16)	0.006		5.73(1.53,21.51)	0.01		0.00(0.00,Inf)	0.996	
oil_fumes			0.188			0.026			0.719			0.194
age_group.1_oil_fume	Ref			Ref			Ref			Ref		

s.1												
age_group.2_oil_fumes.1	1.67(1.00,2.80)	0.052		1.71(1.14,2.56)	0.009		3.88(1.29,11.62)	0.015		inf.(0.00,Inf)	0.982	
age_group.3_oil_fumes.1	2.61(1.51,4.51)	<0.001		2.07(1.35,3.19)	<0.001		9.77(3.39,28.23)	<0.001		5212727.52(0.00,Inf)	0.983	
age_group.1_oil_fumes.2	2.61(1.78,3.81)	<0.001		2.27(1.53,3.37)	<0.001		1.34(0.39,4.65)	0.642		1436207.50(0.00,Inf)	0.985	
age_group.2_oil_fumes.2	2.64(1.62,4.32)	<0.001		2.14(1.49,3.07)	<0.001		4.74(1.69,13.31)	0.003		inf.(0.00,Inf)	0.982	
age_group.3_oil_fumes.2	4.11(2.45,6.91)	<0.001		2.82(1.93,4.11)	<0.001		16.00(5.79,44.22)	<0.001		inf.(0.00,Inf)	0.982	
age_group.1_oil_fumes.3	4.82(2.15,10.81)	<0.001		4.05(1.74,9.42)	0.001		0.00(0.00,Inf)	0.976		1.97(0.00,Inf)	1	
age_group.2_oil_fumes.3	5.45(2.87,10.32)	<0.001		3.17(1.77,5.69)	<0.001		8.94(2.62,30.56)	<0.001		inf.(0.00,Inf)	0.981	
age_group.3_oil_fumes.3	13.80(7.25,26.28)	<0.001		5.72(3.29,9.93)	<0.001		19.89(6.42,61.63)	<0.001		inf.(0.00,Inf)	0.981	
age_group.1_oil_fumes.4	9.11(1.14,32.80)	0.037		0.69(0.04,11.45)	0.798		0.00(0.00,Inf)	0.989		inf.(0.00,Inf)	0.981	
age_group.2_oil_fumes.4	8.94(3.05,26.23)	<0.001		2.91(0.92,9.27)	0.07		7.39(1.03,53.22)	0.047		inf.(0.00,Inf)	0.979	
age_group.3_oil_fumes.4	13.74(3.31,35.99)	<0.001		11.42(2.97,43.90)	<0.001		34.85(6.28,193.31)	<0.001		inf.(0.00,Inf)	0.982	
dm			0.002			0.02			0.631			0.398
age_group.1_dm.0	Ref			Ref			Ref			Ref		
age_group.2_dm.0	1.34(0.89,2.01)	0.157		1.18(0.94,1.49)	0.148		3.84(1.90,7.79)	<0.001		23.25(3.11,173.92)	0.002	
age_group.3_dm.0	2.04(1.32,3.15)	0.001		1.45(1.13,1.86)	0.003		11.10(5.56,22.16)	<0.001		18.89(2.48,143.84)	0.005	
age_group.1_dm.1	3.05(1.92,4.86)	<0.001		1.99(1.20,3.31)	0.007		1.24(0.25,6.14)	0.788		3.92(0.19,79.58)	0.374	
age_group.2_dm.1	2.66(1.61,4.38)	<0.001		1.50(1.01,2.25)	0.047		6.05(2.56,14.28)	<0.001		8.82(0.85,91.77)	0.068	
age_group.3_dm.1	10.65(5.90,19.21)	<0.001		4.13(2.54,6.70)	<0.001		23.92(10.38,55.13)	<0.001		8.34(0.72,96.68)	0.09	
natural_gas			0.407			0.336			0.65			0.917
age_group.1_natural_gas.0	Ref			Ref			Ref			Ref		
age_group.2_natural_gas.0	1.48(0.82,2.66)	0.192		1.44(0.86,2.42)	0.165		2.46(0.76,8.04)	0.135		9.08(0.98,84.13)	0.052	
age_group.3_natural_gas.0	2.64(1.48,4.71)	0.001		2.06(1.27,3.33)	0.003		8.83(3.02,25.82)	<0.001		8.16(0.91,73.23)	0.061	
age_group.1_natural_gas.1	1.07(0.71,1.63)	0.746		1.18(0.75,1.85)	0.466		0.50(0.14,1.77)	0.28		0.48(0.03,8.66)	0.618	
age_group.2_natural_g	1.27(0.74,2.16)	0.383		1.24(0.81,1.91)	0.323		2.38(0.83,6.86)	0.107		7.89(0.91,68.30)	0.061	

as.1												
age_group.3_natural_gas.1	2.02(1.16,3.51)	0.013		1.62(1.04,2.53)	0.032		7.02(2.46,20.07)	<0.001		6.22(0.70,55.67)	0.102	
season			<0.001			<0.001			0.209			0.234
age_group.1_season.1	Ref			Ref			Ref			Ref		
age_group.2_season.1	1.58(1.05,2.40)	0.029		1.17(0.88,1.54)	0.281		4.13(1.84,9.27)	<0.001		18.19(2.32,142.67)	0.006	
age_group.3_season.1	1.67(1.06,2.62)	0.026		1.06(0.77,1.46)	0.728		10.13(4.52,22.67)	<0.001		19.54(2.46,155.21)	0.005	
age_group.1_season.2	0.66(0.47,0.93)	0.019		0.54(0.38,0.77)	<0.001		0.50(0.14,1.75)	0.28		1.24(0.07,20.73)	0.883	
age_group.2_season.2	0.66(0.44,1.00)	0.047		0.55(0.40,0.74)	<0.001		1.89(0.80,4.45)	0.146		10.00(1.24,80.51)	0.031	
age_group.3_season.2	1.87(1.19,2.93)	0.007		1.15(0.85,1.57)	0.362		7.85(3.51,17.57)	<0.001		4.57(0.52,39.88)	0.17	
Inhalabie particles and toxic gases			0.023			0.776			0.003			0.353
age_group.1_IPTGl.0	Ref			Ref			Ref			Ref		
age_group.2_IPTGl.0	1.27(0.86,1.89)	0.236		1.11(0.89,1.38)	0.35		4.10(2.10,8.02)	<0.001		20.87(2.84,153.49)	0.003	
age_group.3_IPTG.0	2.20(1.44,3.37)	<0.001		1.51(1.20,1.90)	<0.001		12.81(6.65,24.66)	<0.001		17.78(2.38,132.53)	0.005	
age_group.1_IPTG.1	6.54(1.93,22.14)	0.003		2.76(0.69,11.12)	0.152		5.26(0.46,60.75)	0.184		21.52(0.83,559.36)	0.065	
age_group.2_IPTGl.1	5.30(2.50,11.22)	<0.001		4.21(2.11,8.40)	<0.001		16.21(5.71,46.02)	<0.001		90.14(9.25,878.20)	<0.001	
age_group.3_IPTG.1	5.10(1.19,21.93)	0.029		3.18(0.59,16.97)	0.177		20.11(10.17,37.35)	<0.001		21.53(0.79,583.45)	0.068	

IPTG:Inhalabie particles and toxic gases

Table S14 Subgroup interaction sheet between gender and disease history factors

characteristic	Overall disease			Acute disease			Chronic disease			Other disease		
	OR(95%CI)	P	P for interaction	OR(95%CI)	P	P for interaction	OR(95%CI)	P	P for interaction	OR(95%CI)	P	P for interaction
history_of_RD			0.124			0.74		0.026			0.207	
sex.1_history_of_RD.0	Ref			Ref	0.208		Ref			Ref		
sex.2_history_of_RD.0	0.98(0.79,1.21)	0.839		1.16(0.92,1.45)	<0.001		0.91(0.56,1.47)	0.705		0.32(0.15,0.68)	0.003	
sex.1_history_of_RD.1	17.60(13.50,22.95)	<0.001		4.82(3.65,6.37)	<0.001		9.03(5.93,13.77)	<0.001		1.46(0.74,2.86)	0.271	
sex.2_history_of_RD.1	12.95(9.96,16.84)	<0.001		5.96(4.50,7.88)		0.404	4.10(2.54,6.60)	<0.001		0.92(0.43,1.94)	0.823	
fourt_phb			0.47						0.402			0.625
sex.1_fourt_phb.0	Ref			Ref	0.05		Ref			Ref		
sex.2_fourt_phb.0	0.83(0.69,1.00)	0.053		1.24(1.00,1.53)	<0.001		0.54(0.37,0.79)	0.002		0.40(0.21,0.77)	0.006	
sex.1_fourt_phb.1	9.60(7.24,12.73)	<0.001		3.77(2.76,5.14)	<0.001		1.58(1.04,2.38)	0.031		1.58(0.78,3.22)	0.203	
sex.2_fourt_phb.1	6.96(5.34,9.07)	<0.001		3.89(2.89,5.25)		0.158	1.11(0.72,1.71)	0.646		0.83(0.38,1.85)	0.655	
cough			0.709						0.026			0.953
sex.1_cough.1	Ref			Ref	0.909		Ref			Ref		
sex.2_cough.1	1.12(0.92,1.38)	0.258		1.01(0.81,1.27)	<0.001		0.97(0.62,1.52)	0.884		0.48(0.24,0.98)	0.043	
sex.1_cough.2	4.13(3.27,5.22)	<0.001		1.70(1.28,2.26)	<0.001		3.63(2.34,5.63)	<0.001		1.78(0.90,3.52)	0.096	
sex.2_cough.2	4.24(3.25,5.53)	<0.001		2.61(1.91,3.56)	0.89		1.38(0.79,2.41)	0.26		0.68(0.26,1.76)	0.425	
sex.1_cough.3	10.46(6.12,17.89)	<0.001		1.04(0.56,1.93)	0.214		11.06(5.74,21.31)	<0.001		2.27(0.68,7.56)	0.182	
sex.2_cough.3	7.13(3.40,14.94)	<0.001		1.79(0.72,4.46)	0.015		5.16(1.83,14.59)	0.002		0.66(0.07,6.12)	0.718	
sex.1_cough.4	10.63(5.46,20.70)	<0.001		2.67(1.21,5.90)	0.002		6.65(2.75,16.07)	<0.001		3.87(1.14,13.12)	0.03	
sex.2_cough.4	13.18(5.12,33.89)	<0.001		5.49(1.88,16.05)		0.575	1.65(0.41,6.68)	0.485		2.17(0.38,12.30)	0.381	
CHD			0.484						0.71			0.774
sex.1_CHD.0	Ref			Ref	0.096		Ref			Ref		
sex.2_CHD.0	0.83(0.71,0.97)	0.021		1.17(0.97,1.42)	0.796		0.61(0.44,0.83)	0.002		0.43(0.24,0.75)	0.003	
sex.1_CHD.1	2.07(1.15,3.72)	0.016		0.90(0.39,2.05)	0.334		3.26(1.43,7.46)	0.005		4.39(1.59,12.15)	0.004	
sex.2_CHD.1	2.31(1.29,4.12)	0.005		1.44(0.68,3.05)		0.912	1.58(0.69,3.61)	0.276		2.36(0.75,7.37)	0.14	

Allergy_history			0.179						0.457			0.215
sex.1_Allergy_history.1	Ref			Ref	0.134		Ref			Ref		
sex.2_Allergy_history.1	0.81(0.67,0.97)	0.021		1.18(0.95,1.46)	<0.001		0.55(0.38,0.80)	0.002		0.33(0.16,0.68)	0.003	
sex.1_Allergy_history.2	7.76(5.85,10.29)	<0.001		3.66(2.66,5.02)	<0.001		1.49(0.96,2.31)	0.073		2.03(1.04,3.96)	0.038	
sex.2_Allergy_history.2	4.87(3.81,6.22)	<0.001		4.41(3.34,5.81)		0.13	1.05(0.67,1.64)	0.827		1.30(0.64,2.63)	0.468	
other_CVD			0.48						0.306		0.075	
sex.1_other_CVD.0	Ref			Ref	0.134		Ref			Ref		
sex.2_other_CVD.0	0.83(0.71,0.97)	0.018		1.16(0.96,1.40)	0.582		0.62(0.45,0.84)	0.003		0.35(0.19,0.64)	<0.001	
sex.1_other_CVD.1	4.11(2.12,7.99)	<0.001		0.79(0.33,1.85)	0.036		2.63(1.03,6.67)	0.042		4.93(1.72,14.10)	0.003	
sex.2_other_CVD.1	4.70(2.58,8.57)	<0.001		2.10(1.05,4.20)			0.78(0.27,2.24)	0.65		6.16(2.54,14.96)	<0.001	

Table S15 Subgroup interaction sheet between age group and disease history factors

characteristic	Overall disease			Acute disease			Chronic disease			Other disease		
	OR(95%CI)	P	P for interaction	OR(95%CI)	P	P for interaction	OR(95%CI)	P	P for interaction	OR(95%CI)	P	P for interaction
history_of_RD			<0.001			<0.001			<0.001			0.954
age_group.1_history_of_RD.0	Ref			Ref			Ref			Ref		
age_group.2_history_of_RD.0	1.57(0.98,2.52)	0.063		1.53(1.15,2.05)	0.004		4.04(1.71,9.53)	0.001		11.76(1.59,87.28)	0.016	
age_group.3_history_of_RD.0	1.25(0.74,2.11)	0.41		1.44(1.02,2.03)	0.04		3.31(1.29,8.47)	0.013		8.55(1.07,68.49)	0.043	
age_group.1_history_of_RD.1	14.89(6.20,28.22)	<0.001		12.87(8.13,20.38)	<0.001		3.33(0.97,11.45)	0.056		2.76(0.16,48.49)	0.488	
age_group.2_history_of_RD.1	6.55(3.57,10.81)	<0.001		5.96(4.23,8.40)	<0.001		13.24(5.47,32.05)	<0.001		20.56(2.64,160.15)	0.004	
age_group.3_history_of_RD.1	20.11(10.31,35.70)	<0.001		5.97(4.16,8.55)	<0.001		41.98(17.79,99.10)	<0.001		15.71(1.97,124.93)	0.009	
fourt_phb			0.001			0.896			0.886			0.246
age_group.1_fourt_phb.0	Ref			Ref			Ref			Ref		
age_group.2_fourt_phb.0	1.20(0.78,1.86)	0.402		1.10(0.84,1.45)	0.491		3.52(1.50,8.29)	0.004		9.59(1.29,71.19)	0.027	
age_group.3_fourt_phb.0	1.76(1.11,2.81)	0.017		0.99(0.72,1.36)	0.962		6.89(2.92,16.25)	<0.001		9.43(1.23,72.46)	0.031	
age_group.1_fourt_phb.1	7.34(5.06,10.66)	<0.001		3.38(2.23,5.12)	<0.001		1.47(0.43,5.06)	0.542		2.34(0.14,40.02)	0.558	
age_group.2_fourt_phb.1	8.34(5.15,13.49)	<0.001		3.70(2.54,5.38)	<0.001		7.12(2.84,17.86)	<0.001		25.54(3.25,200.56)	0.002	
age_group.3_fourt_phb.1	18.69(6.08,31.18)	<0.001		3.73(2.43,5.73)	<0.001		12.80(5.15,31.77)	<0.001		9.80(1.12,85.70)	0.039	
cough			<0.001			0.119			0.106			0.596
age_group.1_cough.1	Ref			Ref			Ref			Ref		
age_group.2_cough.1	1.16(0.76,1.78)	0.489		1.11(0.84,1.46)	0.462		3.65(1.70,7.81)	<0.001		12.19(1.63,90.94)	0.015	
age_group.3_cough.1	1.27(0.80,2.03)	0.313		1.20(0.87,1.67)	0.273		4.51(2.01,10.13)	<0.001		9.87(1.23,79.21)	0.031	
age_group.1_cough.2	3.67(2.41,5.60)	<0.001		2.82(1.73,4.58)	<0.001		1.25(0.26,6.09)	0.781		0.00(0.00,Inf)	0.983	
age_group.2_cough.2	3.50(2.23,5.48)	<0.001		2.43(1.75,3.37)	<0.001		5.30(2.34,12.02)	<0.001		25.34(3.31,194.09)	0.002	
age_group.3_cough.2	6.96(4.30,11.25)	<0.001		1.79(1.25,2.55)	0.001		14.48(6.73,31.16)	<0.001		12.89(1.59,104.68)	0.017	
age_group.1_cough.3	1.16(0.26,5.19)	0.844		0.61(0.10,3.71)	0.591		5.58(0.58,53.41)	0.136		0.00(0.00,Inf)	0.994	

age_group.2_cough.3	7.66(3.38,17.34)	<0.001		1.12(0.46,2.74)	0.796		22.95(7.56,69.70)	<0.001		14.10(0.82,243.43)	0.069	
age_group.3_cough.3	12.37(3.14,22.26)	<0.001		1.60(0.80,3.23)	0.186		42.90(16.63,110.62)	<0.001		28.77(2.91,284.29)	0.004	
age_group.1_cough.4	6.98(2.27,21.47)	<0.001		1.48(0.35,6.25)	0.596		0.00(0.00,Inf)	0.974		26.41(1.25,558.85)	0.036	
age_group.2_cough.4	11.35(4.87,26.47)	<0.001		6.75(2.88,15.83)	<0.001		10.28(2.65,39.85)	<0.001		34.14(2.74,425.07)	0.006	
age_group.3_cough.4	16.42(7.38,30.81)	<0.001		2.23(0.70,7.11)	0.174		57.22(15.44,212.14)	<0.001		66.81(6.04,739.03)	<0.001	
CHD			0.574			0.919			0.575			0.071
age_group.1_CHD.0	Ref			Ref			Ref			Ref		
age_group.2_CHD.0	1.23(0.83,1.82)	0.296		1.10(0.87,1.39)	0.42		3.99(2.07,7.69)	<0.001		9.19(2.15,39.20)	0.003	
age_group.3_CHD.0	1.99(1.31,3.04)	0.001		1.02(0.78,1.34)	0.875		7.81(4.04,15.08)	<0.001		8.28(1.86,36.81)	0.005	
age_group.1_CHD.1	0	NA		0.00(0.00,inf.)	0.976		0.00(0.00,inf.)	0.983		0.00(0.00,Inf)	0.99	
age_group.2_CHD.1	4.00(1.28,8.50)	0.017		1.41(0.32,6.26)	0.649		17.08(2.96,98.48)	0.001		179.56(25.19,1280.06)	<0.001	
age_group.3_CHD.1	4.00(2.20,7.27)	<0.001		1.11(0.60,2.06)	0.747		12.09(5.12,28.56)	<0.001		19.41(3.60,104.78)	<0.001	
Allergy_history			<0.001			<0.001			0.956			0.729
age_group.1_Allergy_history.1	Ref			Ref			Ref			Ref		
age_group.2_Allergy_history.1	1.66(1.05,2.62)	0.031		1.51(1.14,2.00)	0.004		4.30(1.92,9.64)	<0.001		12.88(1.73,95.68)	0.013	
age_group.3_Allergy_history.1	2.60(1.61,4.21)	<0.001		1.42(1.04,1.94)	0.026		8.01(3.60,17.83)	<0.001		8.28(1.06,64.58)	0.044	
age_group.1_Allergy_history.2	10.25(3.90,21.80)	<0.001		12.32(7.52,20.17)	<0.001		1.95(0.53,7.13)	0.313		3.85(0.22,67.41)	0.355	
age_group.2_Allergy_history.2	7.31(4.54,11.77)	<0.001		4.58(3.30,6.34)	<0.001		6.99(3.00,16.28)	<0.001		24.92(3.25,190.86)	0.002	
age_group.3_Allergy_history.2	14.46(8.05,29.24)	<0.001		3.40(2.14,5.41)	<0.001		13.99(5.81,33.69)	<0.001		23.42(2.85,192.69)	0.003	
other_CVD			0.145			0.655			0.38			0.655
age_group.1_other_CVD.0	Ref			Ref			Ref			Ref		
age_group.2_other_CVD.0	1.29(0.86,1.91)	0.215		1.11(0.87,1.40)	0.4		4.39(2.23,8.67)	<0.001		8.38(2.00,35.20)	0.004	
age_group.3_other_CVD.0	2.11(1.38,3.23)	<0.001		1.03(0.79,1.36)	0.809		8.04(4.06,15.91)	<0.001		5.91(1.32,26.33)	0.02	
age_group.1_other_CVD.1	0	NA		3.66(0.27,49.44)	0.329		6.93(0.42,113.88)	0.175		0.00(0.00,Inf)	0.985	
age_group.2_other_CV	4.95(2.50,9.79	<0.001		1.49(0.74,3.01)	0.264		4.41(1.23,15.83)	0.023		60.97(12.11,306	<0.00	

D.1	)									.94)	1	
age_group.3_other_CVD.1	12.25(5.02,29.93)	<0.001		1.08(0.44,2.67)	0.869		16.75(5.39,52.04)	<0.001		56.66(9.91,323.93)	<0.001	

Table S16 Subgroup interaction sheet between gender and family history factors

characteristic	Overall disease			Acute disease			Chronic disease			Other disease		
	OR(95%CI)	P	P for interaction	OR(95%CI)	P	P for interaction	OR(95%CI)	P	P for interaction	OR(95%CI)	P	P for interaction
COPD_f			0.568			0.161			0.62			0.039
sex.1_COPD_f.0	Ref			Ref			Ref			Ref		
sex.2_COPD_f.0	0.82(0.69,0.97)	0.021		0.99(0.83,1.19)	0.949		0.48(0.34,0.68)	<0.001		0.27(0.13,0.55)	<0.001	
sex.1_COPD_f.1	6.86(5.07,9.30)	<0.001		1.93(1.37,2.73)	<0.001		2.27(1.47,3.51)	<0.001		1.63(0.76,3.50)	0.209	
sex.2_COPD_f.1	6.32(4.73,8.44)	<0.001		2.64(1.91,3.67)	<0.001		0.93(0.57,1.51)	0.766		1.33(0.61,2.90)	0.476	
Bronchitis_f			0.151			0.043			0.316			0.596
sex.1_Bronchitis_f.0	Ref			Ref			Ref			Ref		
sex.2_Bronchitis_f.0	0.79(0.67,0.94)	0.009		0.96(0.80,1.16)	0.701		0.51(0.35,0.72)	<0.001		0.39(0.21,0.73)	0.003	
sex.1_Bronchitis_f.1	4.12(3.13,5.43)	<0.001		1.34(0.96,1.87)	0.083		2.36(1.56,3.56)	<0.001		0.92(0.44,1.93)	0.823	
sex.2_Bronchitis_f.1	4.32(3.31,5.63)	<0.001		2.00(1.48,2.72)	<0.001		0.88(0.55,1.41)	0.588		0.48(0.21,1.12)	0.089	
Eczema_f			0.554			0.004			0.104			0.378
sex.1_Eczema_f.0	Ref			Ref			Ref			Ref		
sex.2_Eczema_f.0	0.83(0.70,0.99)	0.035		0.95(0.79,1.13)	0.56		0.54(0.38,0.77)	<0.001		0.36(0.18,0.70)	0.003	
sex.1_Eczema_f.1	10.16(3.44,20.25)	<0.001		3.11(2.16,4.47)	<0.001		6.92(4.48,10.70)	<0.001		3.80(1.77,8.17)	<0.001	
sex.2_Eczema_f.1	9.78(13.40,29.21)	<0.001		5.95(4.12,8.58)	<0.001		2.28(1.38,3.77)	0.001		2.16(0.92,5.05)	0.077	
Lung_fibrosis_f			0.81			0.764			0.746		0.243	
sex.1_Lung_fibrosis_f.0	Ref			Ref			Ref			Ref		
sex.2_Lung_fibrosis_f.0	0.83(0.71,0.97)	0.017		1.05(0.89,1.25)	0.544		0.45(0.33,0.61)	<0.001		0.38(0.22,0.66)	<0.001	
sex.1_Lung_fibrosis_f.1	10.89(3.19,27.47)	<0.001		2.65(1.31,5.38)	0.007		0.98(0.48,2.01)	0.962		1.20(0.41,3.48)	0.739	
sex.2_Lung_fibrosis_f.1	9.68(2.78,19.61)	<0.001		2.43(1.31,4.52)	0.005		0.51(0.25,1.04)	0.066		1.04(0.39,2.79)	0.942	

Table S17 Subgroup interaction sheet between age group and family history factors

characteristic	OR(95%CI)	P	P for interaction	OR(95%CI)	P	P for interaction	OR(95%CI)	P	P for interaction	OR(95%CI)	P	P for interaction
COPD_f			0.723			0.044			0.758			0.578
age_group.1_COPD_f.0	Ref			Ref			Ref			Ref		
age_group.2_COPD_f.0	1.23(0.81,1.87)	0.333		1.09(0.86,1.37)	0.483		3.18(1.56,6.48)	0.002		14.76(2.00,108.97)	0.008	
age_group.3_COPD_f.0	2.13(1.36,3.35)	<0.001		1.38(1.07,1.78)	0.012		9.37(4.66,18.83)	<0.001		15.95(2.11,120.82)	0.007	
age_group.1_COPD_f.1	8.37(4.79,14.63)	<0.001		4.20(2.31,7.62)	<0.001		1.57(0.32,7.72)	0.581		10.18(0.61,170.50)	0.107	
age_group.2_COPD_f.1	9.34(5.76,15.15)	<0.001		2.50(1.73,3.59)	<0.001		5.58(2.50,12.48)	<0.001		30.82(3.84,247.63)	0.001	
age_group.3_COPD_f.1	13.98(8.15,23.99)	<0.001		2.36(1.57,3.55)	<0.001		20.56(9.51,44.45)	<0.001		33.47(4.09,274.21)	0.001	
Bronchitis_f			0.046			0.087			0.451			0.016
age_group.1_Bronchitis_f.0	Ref			Ref			Ref			Ref		
age_group.2_Bronchitis_f.0	1.30(0.86,1.97)	0.212		1.11(0.88,1.40)	0.367		3.60(1.70,7.62)	<0.001		inf.(0.00,Inf)	0.975	
age_group.3_Bronchitis_f.0	2.05(1.31,3.21)	0.002		1.20(0.93,1.55)	0.164		9.93(4.75,20.75)	<0.001		inf.(0.00,Inf)	0.975	
age_group.1_Bronchitis_f.1	4.78(2.87,7.98)	<0.001		2.21(1.26,3.89)	0.006		2.27(0.57,9.09)	0.247		9168676.37(0.00,Inf)	0.976	
age_group.2_Bronchitis_f.1	5.23(3.28,8.32)	<0.001		1.49(1.06,2.11)	0.023		5.59(2.46,12.75)	<0.001		9333783.35(0.00,Inf)	0.976	
age_group.3_Bronchitis_f.1	13.89(8.20,23.54)	<0.001		2.59(1.74,3.84)	<0.001		22.87(10.30,50.77)	<0.001		inf.(0.00,Inf)	0.975	
Eczema_f			0.837			0.031			0.229			0.027
age_group.1_Eczema_f.0	Ref			Ref			Ref			Ref		
age_group.2_Eczema_f.0	1.20(0.77,1.85)	0.419		1.07(0.86,1.35)	0.54		4.20(1.90,9.28)	<0.001		inf.(0.00,Inf)	0.975	
age_group.3_Eczema_f.0	1.90(1.19,3.03)	0.007		1.37(1.07,1.76)	0.012		11.36(5.20,24.82)	<0.001		inf.(0.00,Inf)	0.975	
age_group.1_Eczema_f.1	25.77(11.82,56.16)	<0.001		9.49(4.46,20.22)	<0.001		9.10(2.46,33.76)	<0.001		inf.(0.00,Inf)	0.974	
age_group.2_Eczema_f.1	24.46(14.11,42.39)	<0.001		4.91(3.31,7.29)	<0.001		15.42(6.36,37.38)	<0.001		inf.(0.00,Inf)	0.973	
age_group.3_Eczema_f.1	28.10(19.79,65.36)	<0.001		4.42(2.92,6.70)	<0.001		65.90(28.28,153.59)	<0.001		inf.(0.00,Inf)	0.973	
Lung_fibrosis_f			0.046			0.16			0.188			0.004
age_group.1_Lung_fibro	Ref			Ref			Ref			Ref		

sis_f.0												
age_group.2_Lung_fibrosis_f.0	1.21(0.81,1.83)	0.351		1.04(0.84,1.30)	0.697		3.11(1.63,5.92)	<0.001		18.76(2.57,137.04)	0.004	
age_group.3_Lung_fibrosis_f.0	1.95(1.26,3.02)	0.003		1.27(1.00,1.61)	0.046		9.20(4.89,17.32)	<0.001		15.14(2.02,113.72)	0.008	
age_group.1_Lung_fibrosis_f.1	13.28(7.23,24.56)	<0.001		11.85(1.38,101.74)	0.024		0.00(0.00,Inf)	0.978		30.22(1.65,554.50)	0.022	
age_group.2_Lung_fibrosis_f.1	18.89(9.18,38.85)	<0.001		2.65(1.39,5.05)	0.003		2.60(1.01,6.66)	0.047		9.86(0.95,101.83)	0.055	
age_group.3_Lung_fibrosis_f.1	21.86(7.59,37.47)	<0.001		2.22(1.03,4.77)	0.042		15.86(6.15,40.91)	<0.001		65.47(7.38,580.97)	<0.001	

Table S18 Subgroup interaction sheet between gender and other factors

characteristic	Overall disease			Acute disease			Chronic disease			Other disease		
	OR(95%CI)	P	P for interaction	OR(95%CI)	P	P for interaction	OR(95%CI)	P	P for interaction	OR(95%CI)	P	P for interaction
emotion			0.372			0.354			0.113			0.578
sex.1_emotion.1	Ref			Ref			Ref			Ref		
sex.2_emotion.1	0.79(0.57,1.08)	0.132		1.02(0.73,1.44)	0.892		0.41(0.19,0.87)	0.021		0.51(0.20,1.31)	0.159	
sex.1_emotion.2	2.17(1.71,2.75)	<0.001		1.79(1.37,2.35)	<0.001		2.16(1.40,3.35)	<0.001		1.02(0.54,1.94)	0.945	
sex.2_emotion.2	1.53(1.21,1.95)	<0.001		1.63(1.25,2.13)	<0.001		0.97(0.61,1.55)	0.907		0.34(0.16,0.75)	0.007	
sex.1_emotion.3	3.73(2.43,5.72)	<0.001		3.46(2.17,5.53)	<0.001		4.27(2.24,8.15)	<0.001		0.33(0.04,2.56)	0.29	
sex.2_emotion.3	2.75(1.94,3.89)	<0.001		2.72(1.87,3.97)	<0.001		0.86(0.41,1.80)	0.694		0.48(0.14,1.69)	0.253	
sex.1_emotion.4	3.27(1.73,6.19)	<0.001		1.85(0.88,3.90)	0.105		1.75(0.60,5.08)	0.305		3.09(0.94,10.21)	0.064	
sex.2_emotion.4	4.55(2.80,7.39)	<0.001		3.41(2.02,5.75)	<0.001		2.00(0.88,4.57)	0.1		1.71(0.54,5.43)	0.36	

Table S19 Subgroup interaction sheet between age group and other factors

characteristic	Overall disease			Acute disease			Chronic disease			Other disease		
	OR(95%CI)	P	P for interaction	OR(95%CI)	P	P for interaction	OR(95%CI)	P	P for interaction	OR(95%CI)	P	P for interaction
emotion			0.394			0.684			0.814			0.042
age_group.1_emotion.1	Ref			Ref			Ref			Ref		
age_group.2_emotion.1	1.47(0.85,2.54)	0.165		1.02(0.67,1.55)	0.916		2.30(0.71,7.43)	0.163		inf.(0.00,Inf)	0.982	
age_group.3_emotion.1	2.32(1.34,4.03)	0.003		1.46(0.97,2.21)	0.07		7.82(2.68,22.84)	<0.001		inf.(0.00,Inf)	0.982	
age_group.1_emotion.2	2.11(1.45,3.08)	<0.001		1.82(1.24,2.66)	0.002		1.21(0.32,4.55)	0.779		0.91(0.00,Inf)	1	
age_group.2_emotion.2	2.68(1.62,4.42)	<0.001		1.77(1.27,2.46)	<0.001		4.47(1.60,12.46)	0.004		inf.(0.00,Inf)	0.982	
age_group.3_emotion.2	5.11(3.03,8.63)	<0.001		2.30(1.61,3.27)	<0.001		15.98(5.79,44.10)	<0.001		inf.(0.00,Inf)	0.982	
age_group.1_emotion.3	5.41(2.97,9.88)	<0.001		4.79(2.61,8.79)	<0.001		3.29(0.58,18.80)	0.18		inf.(0.00,Inf)	0.982	
age_group.2_emotion.3	4.21(2.36,7.50)	<0.001		2.58(1.64,4.04)	<0.001		5.27(1.65,16.83)	0.005		8240651.19(0.00,Inf)	0.983	
age_group.3_emotion.3	8.90(4.60,17.22)	<0.001		4.34(2.48,7.60)	<0.001		21.38(6.81,67.16)	<0.001		0.61(0.00,Inf)	1	
age_group.1_emotion.4	4.67(1.65,13.24)	0.004		3.24(1.07,9.80)	0.037		0.00(0.00,inf.)	0.973		inf.(0.00,Inf)	0.981	
age_group.2_emotion.4	7.50(3.97,14.16)	<0.001		3.11(1.77,5.47)	<0.001		8.64(2.53,29.50)	<0.001		inf.(0.00,Inf)	0.981	
age_group.3_emotion.4	6.30(2.46,16.13)	<0.001		3.11(1.23,7.87)	0.017		15.75(3.70,66.95)	<0.001		inf.(0.00,Inf)	0.981	
